# Co-occurrence across time and space of drug- and cannabinoid- exposure and adverse mental health outcomes in the National Survey of Drug Use and Health: combined geotemporospatial and causal inference analysis

**DOI:** 10.1186/s12889-020-09748-5

**Published:** 2020-11-04

**Authors:** Albert Stuart Reece, Gary Kenneth Hulse

**Affiliations:** 1 Department of Psychiatry, University of Western Australia, Crawley, Western Australia Australia; 2 Department of Health Sciences, Edith Cowan University, Joondalup, Western Australia Australia

**Keywords:** Cannabis, Cannabinoid, Δ9-tetrahydrocannabinol, Cannabigerol, Mental illness, Major depressive illness, Suicidal ideation, Pathways and mechanisms

## Abstract

**Abstract:**

Background: Whilst many studies have linked increased drug and cannabis exposure to adverse mental health (MH) outcomes their effects on whole populations and geotemporospatial relationships are not well understood.

**Methods:**

Ecological cohort study of National Survey of Drug Use and Health (NSDUH) geographically-linked substate-shapefiles 2010–2012 and 2014–2016 supplemented by five-year US American Community Survey. Drugs: cigarettes, alcohol abuse, last-month cannabis use and last-year cocaine use. MH: any mental illness, major depressive illness, serious mental illness and suicidal thinking. Data analysis: two-stage, geotemporospatial, robust generalized linear regression and causal inference methods in R.

**Results:**

410,138 NSDUH respondents. Average response rate 76.7%. When drug and sociodemographic variables were combined in geospatial models significant terms including tobacco, alcohol, cannabis exposure and various ethnicities remained in final models for all four major mental health outcomes. Interactive terms including cannabis were related to any mental illness (β-estimate = 1.97 (95%C.I. 1.56–2.37), *P* <  2.2 × 10^− 16^), major depressive episode (β-estimate = 2.03 (1.54–2.52), *P* = 3.6 × 10^− 16^), serious mental illness (SMI, β-estimate = 2.04 (1.48–2.60), *P* = 1.0 × 10^− 12^), suicidal ideation (β-estimate = 1.99 (1.52–2.47), *P* <  2.2 × 10^− 16^) and in each case cannabis alone was significantly associated (from β-estimate = − 3.43 (− 4.46 − −2.42), *P* = 3.4 × 10^− 11^) with adverse MH outcomes on complex interactive regression surfaces. Geospatial modelling showed a monotonic upward trajectory of SMI which doubled (3.62 to 7.06%) as cannabis use increased. Extrapolated to whole populations cannabis decriminalization (4.26%, (4.18, 4.34%)), Prevalence Ratio (PR) = 1.035(1.034–1.036), attributable fraction in the exposed (AFE) = 3.28%(3.18–3.37%), *P* < 10^− 300^) and legalization (4.75% (4.65, 4.84%), PR = 1.155 (1.153–1.158), AFE = 12.91% (12.72–13.10%), P < 10^− 300^) were associated with increased SMI vs. illegal status (4.26, (4.18–4.33%)).

**Conclusions:**

Data show all four indices of mental ill-health track cannabis exposure across space and time and are robust to multivariable adjustment for ethnicity, socioeconomics and other drug use. MH deteriorated with cannabis legalization. Cannabis use-MH data are consistent with causal relationships in the forward direction and include dose-response and temporal-sequential relationships. Together with similar international reports and numerous mechanistic studies preventative action to reduce cannabis use is indicated.

**Supplementary Information:**

**Supplementary information** accompanies this paper at 10.1186/s12889-020-09748-5.

## Background

It is widely understood that the use of addictive substances impacts mental health adversely. Cannabis use has been linked with numerous adverse mental health outcomes including reduced educational achievement [1, 2], increased criminal involvement [3], reduced accomplishment of adult goals (education, employment, stable long term relationships) [4], depression [5–7] bipolar disorder [8–10], anxiety [7, 11–13], suicidality [7, 10, 14, 15], schizophrenia, psychosis [16–23] and other drug use [24].

Indeed one notes that the existence and mission of the US Substance Abuse and Mental Health Services Administration (SAMHSA) aims to minimize the incidence of both substance abuse and mental ill-health in order to advance the behavioural health of the nation [25] and that of the National Institute of Drug Abuse is not dissimilar [26]. As such it is widely perceived that substance use may negatively impact major mental health outcomes. This issue was clearly crystallized by the Director of the SAMHSA, Dr. Elinore McCantz-Katz in her presentation of the 2017 National Survey of Drug Use and Health (NSDUH) results which showed in a nationally representative sample of 18–25 year old young adults from 2008 to 2017, a doubling of serious mental health issues from 3.8 to 7.5% and of suicidal plans from 2.0 to 3.7% in the context of past month cannabis use rates rising from 17.3 to 22.1% but falling use of tobacco and alcohol products and low use rates of opioids and cocaine use [27].

This implies that the unbridled adoption of the widespread use of new addictive psychoactive substances may potentially have far-reaching psychological implications with possible impacts at the public health level. It would appear inevitable that in view of the known adverse effects of cannabis on mental health at the molecular, cellular and epidemiological levels [28–33] its widespread deployment in the community would necessarily be causally linked with numerous indices of deteriorating mental health. This was of particular concern in USA in view of the appalling deterioration in the mental health of young adults described in detail by SAMHSA (above paragraph).

In the present context this applies particularly to cannabis use which, since the takeover of various cannabis operations by major tobacco corporations, seems poised at the threshold of major commercialization and global launch by utilizing the global reach and marketing platform of what is popularly known as the “Big Tobacco” industry. Notwithstanding its representation in popular culture as a relatively harmless “soft” drug, cannabis use has been shown to be linked with a variety of negative mental health outcomes including cannabis dependency and use disorder, an impaired lifetime trajectory and fulfilment of adult goals, an amotivational state, an increased incidence of graduation to use of other addictive agents, depression, anxiety, bipolar disorder, schizophrenia and suicide [2, 4, 17, 24, 28, 34–40].

Such being the case one would expect patterns of mental health to follow cannabis use across both time and space. The NSDUH conducted annually by SAMHSA is a globally unique drug dependency and mental health research resource which allows investigation of both substance use and mental health at relatively high spatial and temporal resolution. Its availability publicly together with the presence of high definition substate shapefiles which link these parameters geospatially at defined time points, and which can be matched with other datasets such as those available through the US census, presents a globally unique opportunity to conduct an important public health investigation of these potentially related trends.

The hypothesis driving the present ecological epidemiological investigation was firstly, that substance use and mental health are linked in a formally demonstrable manner, and secondly, that increasing rates of cannabis use would be reflected at the level of population health trends in a robust manner which persisted after adjustment for other common sociodemographic variables. These hypotheses was formulated prior to study commencement. We considered that it was important to use modern geospatiotemporal regression and the tools of formal causal inference in investigating these questions and associations, and in particular in assessing the potentially causal nature of the relationship. A corollary of this is that one might expect metrics of mental health to be worse in states where cannabis is legal. This hypothesis was also tested.

Whilst a link between substance use and adverse mental health is well described in various clinical contexts what is not clear is the extent to which the mental health of whole populations is impacted with particular reference to trends across both space and time and considerations of a putatively causal relationship. It was these gaps that the present research aimed to fill.

It follows that such an enquiry is particularly timely at the present juncture given what appears to be a clear and present international threat to global mental health. On the international scene cannabis is clearly enjoying a modern renaissance under its falsely reassuring image as a low toxicity compound. If concerning trends can be identified and described in USA then it follows that such concerns are likely to apply elsewhere, most particularly if the causal nature of the relationship could be demonstrated at the population health level. For these reasons the present study was timely and important not only for the health of Americans, but indeed to protect the global community of nations.

## Methods

### Data

NSDUH Data on drug use by area was downloaded from the publicly available NSDUH SAMHSA substate shapefiles for 2010–2012 and 2014–2016 [41, 42]. A NSDUH shapefile for 2012–2014 exists but as it substantially overlaps the other two its inclusion would significantly complicate the analysis so this has not been used. This implies that data for 2015 was not used in the present analysis. On occasion the triennia were referred to by their middle year, hence 2011 and 2015. Over 405,000 participants were surveyed across the 6 years. The 2014–2016 shapefile divides the USA into 395 substate areas based either on county or congressional district boundaries. The four drugs of interest were last month cigarette use, past year alcohol abuse or dependence, last month cannabis use, and last year cocaine use which are abbreviated to cigmon, abodalc, mrjmon and cocyr in the NSDUH documentation. These drugs were treated as covariates for cannabis use. Unfortunately no consistent nomenclature for opioid exposure could be identified across both shapefiles. The four mental illnesses mentioned in the NSDUH shapefiles are any mental illness in the past year, major depressive episode, serious mental illness in the past year and suicidal thinking whose NSDUH abbreviations are amiyr, mde, smiyr and suithyr. Serious mental illness is defined as a “mental, behavioural or emotional disorder resulting in severe functional impairment which substantially interferes with one or more major life activities” and includes the diagnoses of major depression, bipolar affective disorder and schizophrenia [43].US Census Bureau County data on ethnicity and median household income (MHY) was downloaded from the via the tidycensus package in R using shapefiles from the R package tigris. Sociodemographic data was derived from the 5 year American Community Survey (ACS, “acs5”) conducted by US Census. The two NSDUH shapefiles were centred on 2011 and 2015 so they were matched to the ACS 2009–2013 and ACS 2013–2017 respectively. Each respective ACS shapefile was then interpolated into the substate area definitions provided by SAMHSA. The two combined NSDUH shapefiles were then combined together with the 2014–2016 NSDUH shapefile as the standard (or “target”) file. Data for Alaska and Hawaii was treated separately and then added in to the final shapefile and elided (moved) into their appropriate positions for illustration purposes.

Data on the concentration of cannabinoids in federal seizures of cannabis to 2011 has been published [44, 45]. In 2011 the concentration of tetrahydrocannabinol (THC) was 11% and it has been increasing by about 1% annually. Projected forwards this provides an estimate of 15% in 2015.

### Statistics

The analysis was conducted in January 2020. Data was processed in “R” from CRAN using several packages including tidyverse, tidycensus, tigris, sp., sf, spdep and splm. Graphs and maps were drawn in ggplot2. Hawaii and Alaska were elided for illustration in sp. (spatial modelling), converted back to sf (simple features) and rendered in the USA contiguous Albers Equal Area Conical projection EPSG:102003 as in the R package albersusa. Disparate geographical boundaries were conformed using R::areal. Statistical model reduction from first to final models was by the classical technique of sequential elimination of the least significant term until only significant terms remain.

The formal analysis of spatially distributed data is not methodologically trivial but requires dedicated methods in order to account for the spatial relationships by which many variables interact and are dependent on each other. In 1970 Waldo Tobler described the first law of geography when he noted that things nearby interact more than things far away [46]. It is important to take this spatial autocorrelation into account when analyzing spatially distributed variables. The package splm in the “R” computing environment is purpose built for such applications. In particular it includes the spatial panel generalized method of moments (spgm) function which is ideally suited to short panel datasets such as this one and the spatial panel random error maximum likelihood (spreml) function which includes sophisticated methods to account for various spatial lag and error structures. Both techniques allow the use of instrumental variables. Thus both techniques have been applied to this dataset.

Geospatiotemporal data processing was done using the “R” package splm (spatial panel linear modelling) with the spatial panel generalized method of moments (spgm) function as it is ideally suited to short panel data such as this and also with the recent spml refinement spreml (spatial panel random error maximum likelihood) function which incorporates sophisticated modelling of error and autocorrelation structures. The standard spgm model was spatially lagged, used the full weights method, a generalized two-step least squared estimation method, spatial error and lagged the instrumental variables. Instrumental variables were the local cannabis ethnic potency index (LCEPI) defined below. For spreml models the full model was used including spatial lagging, spatial errors of Kapoor, Kelejian and Prucha [47], autocorrelation order 1 errors and the same ethnic instrumental variables as above. Spatial errors and spatial weights were calculated using the spdep::poly2nb function and these data were updated to include conceptual links from the Hawaiian islands to south-eastern California and from Alaska to Washington state and Oregon.

Modelling of fitted values was done by matrix multiplication of mean, minimum and maximum values into model coefficients as indicated. Analysis of the impact of cannabis legal status at state level was undertaken from the state-based NSDUH data table (“state_saes_final.sas7bdat”) supplemented by cannabis legal status defined from an internet search. Data was manipulated with dplyr and the resulting two-by-two tables were analyzed in epiR.

### Causal inference analysis

Inverse probability weighting was conducted for the whole sample using the R package ipw. This transforms our study from merely ecological and observational to a pseudo-randomized design where causal inferential relationships can properly be assigned. These weights were then entered into robust generalized linear regression using the R package survey with substate region as the identifying variable. In order to calculate a model standard deviation the weights were also utilized in mixed effects regression using the R package nlme again with substate region as the grouping variable.

The e-Value is a new index which was recently defined [48] which quantitates the degree of association with both the exposure and the outcome which would be required of an unmeasured confounding variable to explain away the observed significant finding [48–53]. It is presented on the risk ratio scale. Research literature commonly contains e-Values of 1.25 and above [52]. e-Values were computed from relative risks and regression coefficients using the R package EValue. *P* < 0.05 was considered significant throughout.

### Data availability statement

Data including shapefiles and R programming script is made publicly available on the Mendeley Data Archive at this URL:

10.17632/gyckst6rx8.1. The original SAMHSA shapefiles may be found at https://www.samhsa.gov/data/report/2014-2016-nsduh-substate-region-shapefile and https://www.samhsa.gov/data/report/2012-2014-nsduh-substate-region-shapefile.

### Ethics

Ethical approval for this study was provided by the University of Western Australia Human Research Ethics Committee 08/01/2020 (No. RA/4/20/4724).

## Results

Of 534,000 individuals approached 410,138 responded to the six NSDUH surveys, a completion rate of 76.7%. Data for the 2 years 2011 and 2015 are listed as median and interquartile ranges and compared non-parametrically in Supplementary Table 1.

**Fig. 1:**
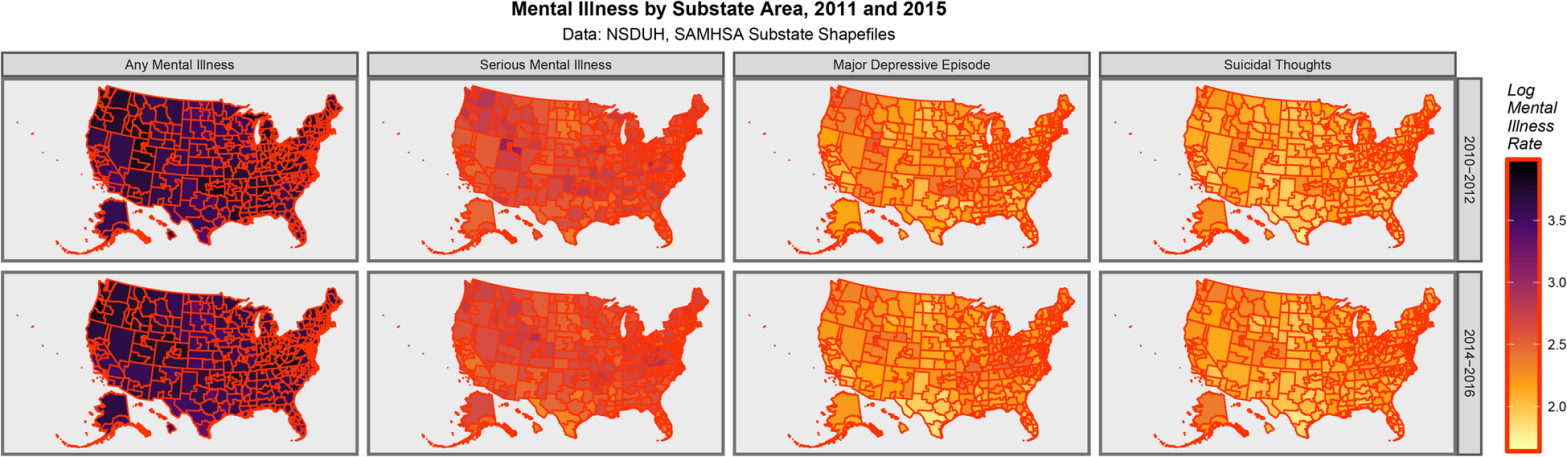
Mental Illness across USA by substate area. Data from NSDUH Shapefiles

Figure 1 shows the rates of mental illness for the four NSDUH-defined mental health disorders included in the SAMHSA substate shapefiles of any mental illness, major depressive episode, serious mental illness and suicidal thinking.

**Fig. 2:**
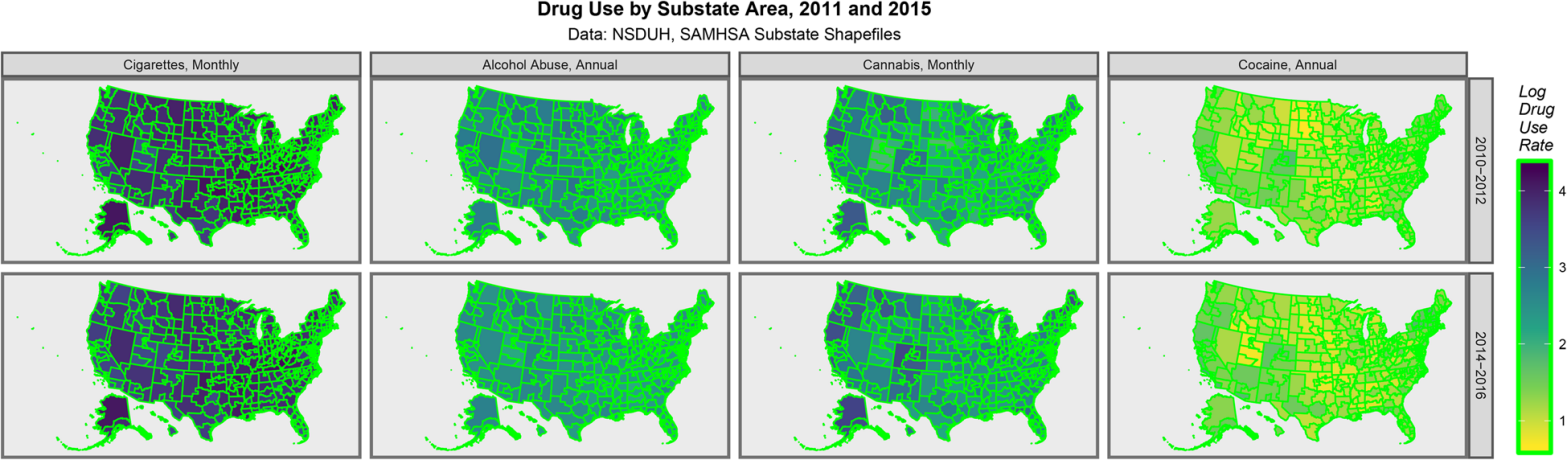
Drug Use across USA by substate area. Data from NSDUH Shapefiles

Figure 2 shows map-graphically the distribution of the use of various drugs across USA in the two NSDUH triennia.

**Fig. 3:**
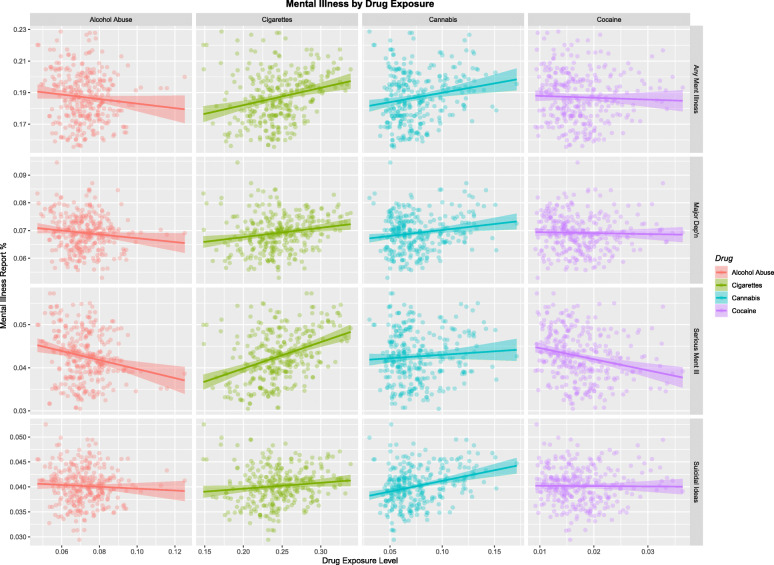
Mental Illness by Substance Exposure. Data from NSDUH Shapefiles

Figure 3 shows the rates of the four mental illness syndromes by drug use at state level. The slope (as β-estimates) and significance of these regression lines is shown in Supplementary Table 2. The slopes for three of the lines is significant.

Supplementary Fig. 1 shows the ethnic composition of USA for the two periods.

Supplementary Fig. 2 shows the rate of median household income in the USA in the two periods 2010–2012 and 2014–2016.

National level NSDUH data make it clear that there are considerable differences between various ethnicities in drug use and especially daily / near daily cannabis use. These can be averaged out by ethnicity to derive a cannabis use frequency index at the national level. It is likely that regional data also impacts cannabis use by ethnic populations so an index of this was derived by multiplying the local monthly cannabis use by the national ethnic near daily cannabis use to derive a local cannabis ethnic daily index (LCEDI) at state level. Since the THC concentration of cannabis has also been increasing the LCEDI can in turn be multiplied by the THC content to produce a local cannabis ethnic daily potency index (LCEDPI) of local ethnic exposure to cannabinoids. This LCEDPI index may also be referred to as an “Ethnic score” and it has been used as an important instrumental variable controlling for environmental cannabinoid exposure arising from the sociocultural environment rather than any intrinsic ethnic risk propensity (such as pharmacogenomic susceptibilities). The various LCEDI and LCEDPI are listed in Supplementary Table 3 and illustrated in Supplementary Figs. 3 and 4. Supplementary Fig. 5 shows the relative rise in these indices from the 2010–2012 baseline and the relative rise comparable to the mean rise by ethnicities.

First degree edge and corner (“queen”) spatial weights were calculated between substate areas by R::spdsep::poly2nb and updated for Alaska, Hawaii and Richmond Island (in New York) as shown in Supplementary Fig. 6A, with final results as shown in Supplementary Fig. 6B.

Supplementary Table 4 presents the spgm results when serious mental illness is regressed against each of drugs, median household income, and ethnicity variables separately with the ethnic LCEPI included as instrumental variables. As noted, only the drug related variables are significant. Terms including cannabis are significant (from β-estimate = 0.08 (95%C.I. 0.02–0.13), *P* = 0.009). Supplementary Table 5 performs the same task for serious mental illness using spreml regression. The significance level of all terms is increased. Both income and racial composition now become significant. Terms including cannabis are significant (from β-estimate = 2.34 (1.71–2.97), *P* = 1.4 × 10^− 13^).

All the independent variables were then included in a final spgm model shown in Table 1 for all four mental illnesses listed by SAMHSA. All four described drugs survive model reduction and appear in final models. The table is notable for the high level of significance of many drugs including terms involving cannabis (from β-estimate = 1.74, (0.97, 2.51), *P* = 9.9 × 10^− 6^). Income and ethnic factors do not survive model reduction. Hence final models include drug related factors only.

**Table 1:** Spatial panel general method of moments regression – final models

General	Parameters			Model		
Instrumental Variables	Parameter	Estimate (95%C.I.)	***P***-Value	Para-meters	Value	***P***-Value
	*Any Mental Illness*					
NHWhite_Score	spgm (amiyr ~ Cigarettes * Cannabis * Alcohol_Abuse + Cocaine + Med_HH_Income + 5_Races)					
NHBlack_Score	Cigarettes: Cannabis: Alcohol_Abuse	−0.05 (− 0.07--0.03)	2.5E-05	rho	− 0.5152	N/A
Hispanic_Score	Cigarettes: Alcohol_Abuse	0.11 (0.06–0.16)	4.6E-05	sigma^2_	0.003	N/A
NHAsian_Score	Cannabis: Alcohol_Abuse	1.21 (0.61–1.81)	7.9E-05	lambda	0.6753	1.30E-05
NHAIAN_Score	Cigarettes: Cannabis	0.09 (0.05–0.13)	0.0001			
	Alcohol_Abuse	−2.43 (−3.72--1.14)	0.0002			
	Cigarettes	−0.17 (− 0.26--0.08)	0.0003			
	Cannabis	−1.99 (−3.08--0.9)	0.0004			
	Cocaine	0.05 (0.01–0.09)	0.0128			
	Median_Household_Income	−0.03 (− 0.06–0)	0.0339			
	Afr.Am_Pop_Fraction	0.03 (0–0.06)	0.0485			
	*Major Depressive Episode*					
NHWhite_Score	spgm (mde ~ Cigarettes * Cannabis * Alcohol_Abuse + Cocaine + Med_HH_Income + 5_Races)					
NHBlack_Score	Cannabis: Alcohol_Abuse	1.74 (0.97–2.51)	9.9E-06	rho	−0.6813	N/A
Hispanic_Score	Cigarettes: Cannabis: Alcohol_Abuse	−0.07 (− 0.1--0.04)	2.6E-05	sigma^2	0.005	N/A
NHAsian_Score	Cannabis	−2.98 (− 4.38--1.58)	2.9E-05	lambda	0.8945	1.20E-04
NHAIAN_Score	Alcohol_Abuse	−3.46 (−5.1--1.82)	3.8E-05			
	Cigarettes: Cannabis	0.12 (0.06–0.18)	6.3E-05			
	Cigarettes: Alcohol_Abuse	0.13 (0.06–0.2)	8.1E-05			
	Cigarettes	−0.23 (− 0.35--0.11)	2.4E-04			
	*Serious Mental Illness*					
NHWhite_Score	spgm (smiyr ~ Cigarettes * Cannabis * Alcohol_Abuse + Cocaine + Med_HH_Income + 5_Races)					
NHBlack_Score	Cigarettes: Alcohol_Abuse	0.1 (0.03–0.17)	0.0048	rho	− 0.7386	N/A
Hispanic_Score	Cigarettes	−0.17 (− 0.29--0.05)	0.0058	sigma^2	0.0055	N/A
NHAsian_Score	Cigarettes: Cannabis: Alcohol_Abuse	−0.04 (− 0.07--0.01)	0.0082	lambda	0.7722	3.37E-07
NHAIAN_Score	Cocaine	0.07 (0.02–0.12)	0.0087			
	Cigarettes: Cannabis	0.08 (0.02–0.14)	0.0092			
	Alcohol_Abuse	−2.14 (−3.82--0.46)	0.0126			
	Cannabis: Alcohol_Abuse	0.96 (0.17–1.75)	0.0169			
	Cannabis	−1.66 (−3.09--0.23)	0.0231			
NHWhite_Score	*Suicidal Thoughts Past Year*					
NHBlack_Score	spgm (suithyr ~ Cigarettes * Cannabis * Alcohol_Abuse + Cocaine + Med_HH_Income + 5_Races)					
Hispanic_Score	Alcohol_Abuse	−3.05 (− 4.67--1.43)	0.0002	rho	−0.6752	N/A
NHAsian_Score	Cannabis: Alcohol_Abuse	1.37 (0.61–2.13)	0.0004	sigma^2_	0.0051	N/A
NHAIAN_Score	Cigarettes: Alcohol_Abuse	0.12 (0.06–0.18)	0.0004	lambda	0.7757	7.96E-12
	Cigarettes: Cannabis: Alcohol_Abuse	−0.05 (− 0.08--0.02)	0.0012			
	Cigarettes	−0.19 (− 0.31--0.07)	0.0016			
	Cannabis	−2.18 (−3.55--0.81)	0.0019			
	Cigarettes: Cannabis	0.08 (0.02–0.14)	0.0037			

Abbreviations

5_Races: Caucasian-American, African-American, Hispanic-American, Asian-American, NHAIAN

Technical Notes:

phi:- Idiosyncratic component of the spatial error term

psi:- Individual time-invariant component of the spatial error term

rho:- Spatial autoregressive parameter

lambda:- Spatial autocorrelation coefficient

Table 2 presents results from a similar exercise applying the advanced techniques of spreml spatial regression. Again all four drugs are included at high level of significance. Terms including cannabis appear (from β-estimate = 1.84 (0.30, 2.39), *P* ≤ 3.0 × 10^− 11^) for all four illness syndromes. Terms including cannabis appear (from β-estimate = − 3.31 (2.58, 4.04), *P* ≤ 2.2 × 10^− 16^ for any mental illness and (from β-estimate = 2.13 (1.63, 2.62), P ≤ 2.2 × 10^− 16^) for major depressive episode. Ethnic factors appear in all models. Median household income only appears in the model for serious mental illness.

**Table 2:** Spatial panel random error maximum likelihood regression – final models

General	Parameters	Model				
Instrumental Variables	Parameter	Estimate (95%C.I.)	***P***-Value	Para-meters	Value	***P***-Value
	*Any Mental Illness*					
	spreml (amiyr ~ Cigarettes * Cannabis * Alcohol_Abuse + Cocaine + Med_HH_Income + 5_Races)					
NHWhite_Score	Alcohol_Abuse	−4.19 (− 5.02--3.36)	< 2.2e-16	phi	0.008	0.9908
NHBlack_Score	Cannabis: Alcohol_Abuse	1.96 (1.55–2.37)	< 2.2e-16	psi	0.4002	0.251
Hispanic_Score	Cannabis	−3.33 (−4.06--2.6)	< 2.2e-16	rho	−0.1507	0.3296
NHAsian_Score	Cigarettes: Alcohol_Abuse	0.15 (0.11–0.19)	< 2.2e-16	lambda	0.2336	0.0425
NHAIAN_Score	Cigarettes	−0.26 (−0.32--0.2)	4.00E-15			
	Cigarettes: Cannabis: Alcohol_Abuse	−0.07 (− 0.09--0.05)	2.70E-15			
	Cigarettes: Cannabis	0.12 (0.09–0.15)	1.20E-13			
	Caucasian-Amer.Pop_Fraction	0.09 (0.06–0.12)	1.00E-10			
	Median_Household_Income	−0.09 (− 0.12--0.06)	1.30E-06			
	Hispanic_Pop_Fraction	−0.01 (− 0.02–0)	0.0033			
	African-Amer._Pop_Fraction	−0.01 (− 0.02–0)	0.0062			
	Asian_Pop_Fraction	0.02 (0.01–0.03)	0.0131			
	***Major Depressive Episode***					
	spreml (mde ~ Cigarettes * Cannabis * Alcohol_Abuse + Cocaine + Med_HH_Income + 5_Races)					
NHWhite_Score	Cannabis: Alcohol_Abuse	2.03 (1.54–2.52)	3.60E-16	phi	0.1573	0.9579
NHBlack_Score	Alcohol_Abuse	−4.14 (−5.14--3.14)	4.90E-16	psi	0.3124	0.8569
Hispanic_Score	Cannabis	−3.53 (− 4.41--2.65)	3.80E-15	rho	−0.3358	0.0325
NHAsian_Score	Cigarettes: Alcohol_Abuse	0.16 (0.12–0.2)	1.60E-12	lambda	0.3809	0.0002
NHAIAN_Score	Cigarettes: Cannabis: Alcohol_Abuse	−0.08 (−0.1--0.06)	1.90E-12			
	Cigarettes	−0.27 (− 0.35--0.19)	4.50E-12			
	Cigarettes: Cannabis	0.14 (0.1–0.18)	5.70E-12			
	Caucasian-Amer._Pop_Fraction	0.08 (0.05–0.11)	2.20E-08			
	Hispanic_Pop_Fraction	−0.02 (−0.03−−0.01)	0.0003			
	African-Amer._Pop_Fraction	−0.01 (− 0.02–0)	0.0025			
	AIAN_Pop_Fraction	-0.01 (−0.02–0)	0.0085			
	Median Household Income	−0.04 (− 0.07--0.01)	0.0312			
	***Serious Mental Illness***					
	spreml (smiyr ~ Cigarettes * Cannabis * Alcohol_Abuse + Cocaine + Med_HH_Income + 5_Races)					
NHWhite_Score	Caucasian-Amer.	0.21 (0.17–0.25)	< 2.2e-16	phi	0.176	NA
NHBlack_Score	Median Household Income	−0.22 (−0.27--0.17)	< 2.2e-16	psi	0.153	NA
Hispanic_Score	Alcohol_Abuse	−4.55 (−5.69--3.41)	6.20E-15	rho	0.1311	0.2141
NHAsian_Score	Cannabis: Alcohol_Abuse	2.04 (1.48–2.6)	1.00E-12	lambda	0.0618	0.4449
NHAIAN_Score	Cannabis	−3.44 (−4.46--2.42)	3.40E-11			
	Cigarettes:Alcohol_Abuse	0.17 (0.12–0.22)	7.60E-11			
	Cigarettes	−0.27 (−0.36--0.18)	1.50E-09			
	Cigarettes: Cannabis: Alcohol_Abuse	−0.08 (− 0.11--0.05)	2.90E-09			
	Cigarettes: Cannabis	0.13 (0.08–0.18)	2.00E-08			
	African-Amer._Pop_Fraction	−0.02 (−0.03--0.01)	9.30E-06			
	Hispanic_Pop_Fraction	−0.02 (− 0.03--0.01)	0.005			
	Asian_Pop_Fraction	0.02 (0–0.04)	0.0089			
	***Suicidal Thoughts Past Year***					
	spreml (suithyr ~ Cigarettes * Cannabis * Alcohol_Abuse + Cocaine + Med_HH_Income + 5_Races)					
NHWhite_Score	Alcohol_Abuse	−4.36 (−5.33--3.39)	< 2.2e-16	phi	0.0225	0.9737
NHBlack_Score	Cannabis: Alcohol_Abuse	1.99 (1.52–2.46)	< 2.2e-16	psi	0.1854	0.726
Hispanic_Score	Cannabis	−3.4 (−4.26--2.54)	1.05E-14	rho	−0.1314	0.2451
NHAsian_Score	Cigarettes: Alcohol_Abuse	0.16 (0.12–0.2)	6.72E-13	lambda	0.2824	0.0005
NHAIAN_Score	Cigarettes	−0.26 (− 0.34--0.18)	8.28E-12			
	Cigarettes: Cannabis: Alcohol_Abuse	−0.07 (− 0.09--0.05)	8.14E-11			
	Cigarettes: Cannabis	0.12 (0.08–0.16)	7.25E-10			
	Hispanic_Pop_Fraction	−0.02 (−0.03--0.01)	5.53E-08			
	African-Amer._Pop_Fraction	−0.02 (− 0.03--0.01)	2.89E-07			
	Caucasian-Amer._Pop_Fraction	0.07 (0.04–0.1)	1.12E-06			
	Asian_Pop_Fraction	0.03 (0.02–0.04)	1.03E-05			
	Median Household Income	−0.08 (−0.12--0.04)	5.70E-05			

Abbreviations

5_Races: Caucasian-American, African-American, Hispanic-American, Asian-American, NHAIAN

Technical Notes:

phi:- Idiosyncratic component of the spatial error term

psi:- Individual time-invariant component of the spatial error term

rho:- Spatial autoregressive parameter

lambda:- Spatial autocorrelation coefficient

As mentioned spreml models give advanced access to the error structure of spatial models. It is therefore mandatory to give careful attention to correct model specification. Supplementary Table 6 shows a selection of the principal error structures and their various specifications. The log maximal likelihood of the models is listed at model optimization, together with the value of the spatial Hausman test comparing each model to the full model. In each case high levels of statistical significance are demonstrated with all *P* <  2.2 × 10^− 16^. These results confirm that the model specification which was presented above, namely the full sem2srre + lag model, is technically correct.

The rates of mental illness can be aggregated into state areas to compare mental illness rates by the legal status of cannabis.

**Fig. 4:**
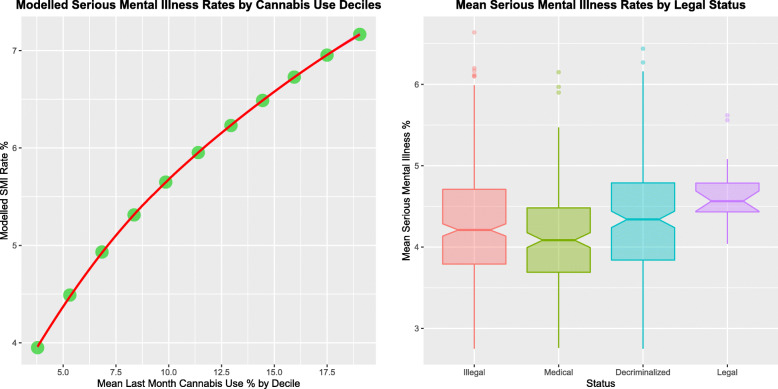
Modelled Relationships and Legal Status. **a** Serious Mental illness by Rising Cannabis Concentration. **b** Serious Mental Illness by Legal Status

The final spreml model for serious mental illness may be used to compute fitted values. When mean values for tobacco and alcohol abuse together with minimum or maximum values for monthly cannabis use are entered into this model minimum and maximum values for serious mental illness of 3.62 and 7.06% result (maximum = 1.95-fold minimum value). Figure 4a shows the modelled rate of serious mental illness as a function of cannabis use decile (Supplementary Table 7).

Data also lend themselves to analysis by the formal techniques of causal inference. Inverse probability weights were calculated as described and entered into robust generalized linear regression equations. The effect of conducting regression procedures with inverse probability weights is to make the sample pseudo-randomly conducted with regard to the exposure of interest, in this case cannabis use, so that the outcome can be properly assessed without the confounding arising from the exposure being non-randomly distributed across the other covariate exposure groups. The results of final models from additive and interactive models as shown in Table 3. In this Table the dependent variable is serious mental illness rates and the list of covariates includes five racial groups, median household income, ethnic cannabis use scores (LCEDI) and drug use variables for cigarettes, binge alcohol, monthly cannabis and annual cocaine. In additive models both cannabis use (β-estimate = − 0.43 (− 0.65 - -0.21), *P* = 0.0002), and Caucasian American cannabis use (β-estimate = 0.95 (0.05–1.85), *P* = 0.0396) survived model reduction and were significant. In models including a four-way cigarette:alcohol:cannabis:cocaine interaction, terms including cannabis were significant from (β-estimate = 0.12 (0.10, 0.14), *P* < 10^− 16^).

**Table 3:** Robust Generalized Linear Models Results

Parameter	Estimate	C.I.	***P***-Value
***Additive Models***			
Cocaine	0.27	(0.13–0.41)	7.6E-05
Alcohol	0.09	(0.05–0.13)	0.0001
Asian.Am	0.12	(0.06–0.18)	0.0005
Median Household Income	0.22	(0.06–0.38)	0.0041
Cauc.Am.Cannabis	0.95	(0.05–1.85)	0.0396
Cannabis	−0.43	(−0.65--0.21)	0.0002
Cigarettes	−0.02	(−0.04–0.00)	6.2E-05
Afric.Am	−0.09	(−0.13--0.05)	5.8E-06
Hispanic	−0.22	(−0.32--0.12)	2.4E-06
***Interactive Models***			
Cigarettes: Alcohol	0.06	(0.04–0.08)	3.2E-26
Cigarettes: Cannabis	0.12	(0.10–0.14)	1.5E-21
Asian.Am	0.07	(0.05–0.09)	4.0E-19
Alcohol: Cannabis	0.51	(0.39–0.63)	2.7E-17
Alcohol: Cannabis: Cocaine	0.04	(0.02–0.06)	2.2E-07
Afric.Am.Cannabis	−0.19	(−0.27--0.11)	3.8E-05
Alcohol: Cocaine	−0.07	(−0.11−− 0.03)	1.9E-05
AIAN.Am	-0.03	(−0.03--0.03)	3.7E-07
Cigarettes: Alcohol: Cannabis	−0.02	(−0.02--0.02)	2.0E-18
Afric.Am	−0.05	(−0.07--0.03)	4.0E-20
Alcohol	−1.41	(−1.65--1.17)	2.0E-26
Cigarettes	−0.30	(−0.36--0.24)	6.4E-28
Cannabis	−2.75	(−3.20--2.30)	3.4E-28

In inverse probability weighted mixed effects additive and interactive models with the same list of dependent variables and predictive covariates, terms including cannabis were again significant (from β-estimate = 0.11 (0.07, 1.15), 1.5 × 10^− 5^; Supplementary Table 8).

Sensitivity analyses may be conducted on these odds ratio, mixed effects and geospatial data with many highly significant e-Value results as shown in Supplementary Table 9. The minimal e-values in the geotemporospatial analyses ranged from 3.13 to 2,660,000 (Supplementary Table 9). This compares positively to comments in the literature that e-Values above 1.25 are often quoted in scientific reports [52]. Such elevated values make uncontrolled confounding extremely unlikely and point to a relationship which is truly causal in nature.

When one considers state-based data for the 6 years of the NSUDH shapefiles states with legal cannabis status had an increased rate of serious mental illness (Prevalence ratio (PR) = 1.09 (95%C.I. 1.04, 1.13), attributable fraction in the exposed (AFE) = 7.93% (4.17, 11.55%), attributable fraction in the population (AFP) = 0.70% (0.035, 1.06%), Chi.Squ. = 16.25, df = 1, *P* = 5.55 × 10^− 5^).

Figure 4b and Table 4 show the mean rate of serious mental illness as a function of cannabis legal status when NSDUH results are extrapolated onto whole state populations. The values for the Illegal, Medical, Decriminalized and Legal Status are 4.26 (4.18, 4.34%), 4.11 (4.01, 4.21%), 4.01 (3.83, 4.19%) and 4.75 (4.65, 4.85%) respectively. The notches for the Decriminalized and Legal statuses are noted to not overlap those of the illegal status. Cannabis decriminalization was associated with an increased incidence of serious mental illness (PR = 1.035 (1.034, 1.036), AFP = 3.28% (3.18, 3.37%), AFE = 1.13% (1.09, 1.16%), ChiSq. = 4635.1, df = 1, P < < 10^− 300^), as was cannabis legalization (PR = 1.155 (1.153, 1.158), AFE = 12.91% (12.72, 13.10%), AFP = 0.83% (0.82, 0.85%), ChiSq. = 15,015.1, df = 1, P < < 10^− 300^).

**Table 4:** Serious mental illness prevalence ratios by legal status

Status	Serious Mental Illness Cases	No. Using Cannabis Last Month	Population	Proportion with Serious Mental Illness	Proportion Using Cannabis Last Month
Decriminalized	6,106,622	12,033,634	143,356,702	4.26% (4.18, 4.34%)	8.39%
Illegal	11,650,796	17,995,589	283,172,529	4.11% (4.01, 4.21%)	6.35%
Legal	805,083	2,337,511	16,936,978	4.75% (4.65, 4.85%)	13.80%
Medical	7,394,236	16,516,827	184,526,713	4.01% (3.83, 4.19%)	8.95%

Supplementary Fig. 7 shows the rate of all mental illness syndromes against cannabis legalization status from state based data. The Chi-squared comparisons are shown in Supplementary Table 10. Results for any mental illness and suicidal ideation are both significant (*P* = 0.0395 and P = 0.0395) are that for serious mental illness approaches significance (*P* = 0.0654).

## Discussion

### Main findings

This study applies current geospatial techniques to the analysis of the four metrics of mental illness spatially described by SAMHSA in recent iterations of NSDUH. Using spatial panel generalized method of moments (spgm) techniques drug-related variables pertaining to tobacco, alcohol, abuse cannabis and cocaine were found to be more significant than socioeconomic and ethnographic factors after correction using estimates for increased local exposure to cannabis in some ethnic groups. For cannabis this included terms significant from *P* < 10^− 5^. When more advanced spatial techniques such as the full spatial panel random error maximum likelihood (spreml) models were used these results were confirmed overall and included an increased level of statistical significance for terms including cannabis for all four mental illness metrics from *P* < 4.0 × 10^− 11^. Therefore geospatial techniques increased the precision of the parameter estimates by several orders of magnitude.

It is of interest to consider these findings in the light of the remarks mentioned in the Introductory section relating to the poor and declining mental health of US young adults. First, there is a very obvious association nationally with the dramatic decline in the mental health of young adults in the USA and rising levels of cannabis use in that age demographic [27, 54]. Our results confirm this trend at the higher geospatial resolution of the substate level.

Secondly both study hypotheses are confirmed by study results. All indices of mental health (any mental illness, major depressive illness, serious mental illness and suicidal ideation) are robustly associated with the use of all addictive substances investigated. It is equally clear that the hypothesized relationships between cannabis and all four indices of mental ill-health are not only established, but robust to multivariable adjustment.

Moreover analysis of the data with inverse probability weights in both mixed effects models and robust generalized linear models together with sensitivity analyses indicated that the relationship fulfilled the criteria of causality in each case.

Any mental health issues and suicidal ideation were also shown to be worse in parallel with liberalized cannabis policies. The result for serious mental illness approached significance (*P* = 0.06).

### Pathways and mechanisms

Since the existence of plausible biological pathways explaining a potential causal pathway from cannabis exposure to mental illness is a foundational pillar of causal algorithms such as that of Hill [55] it becomes very important to consider briefly some of the neurotoxic mechanisms which have been described in the published literature. We note that numerous biological pathways have been described linking cannabinoid exposure to neurotoxicity and adverse neuropsychiatric outcomes. Several genetic and epigenetic pathways have been described linking altered dopamine receptor gene and other gene expression with addictive, behavioural and autistic outcomes [56–60]. Cannabinoids have been shown to have adverse effects on neural stem cell activity [61] which negatively and importantly impacts brain plasticity and brain aging [62]. Cannabinoids can also induce microglial activation and priming [63] which was recently shown to set the brain on a pathway which phenocopies aging [64]. Cannabis exposure has also been shown to age the human organism in a longitudinal study of cardiovascular ageing [64]. Cannabinoids have been shown to decouple both synapses, by negatively impacting the neurexin-neuroligin machinery which scaffolds them [65–68], and grey-white matter coupling [69]. Similarly cannabinoids negatively impact both actin and tubulin expression and dynamics [65] impacting axonal guidance and growth cone mechanics [70] and chromosomal mechanisms, chromosomal segregation and cell division [71]. Cannabis has a negative effect on cell growth, macromolecular synthesis and cell division [72, 73] and adversely affects the slit:robo ratio which controls the hypertrophic exuberant growth of the massive human cerebral cortex [74–76].

It is also important to appreciate that such negative cellular mechanisms have been ascribed to cannabinoids other than simply Δ9-tetrahydrocannabinol as other chemical moieties, including cannabidiol, cannabichromene and cannabinol have been similarly implicated [77–80]. Indeed it is known that cannabis oil is toxic to many plants including the leaves of *Cannabis sativa* itself [81].

It is also relevant in this regard that both the epigenetic actions and chromosomal mis-segregation actions of cannabinoids imply not only genotoxicity and epigenotoxicity in the exposed individuals themselves, but also heritable changes to several subsequent generations [82]. As the use of cannabis becomes both more widespread and consumption increases in existing users, cannabinoid exposure will likely become multigenerational and open new routes to cannabinoid-induced heritable neuropsychopathology. This was recently shown for autism in USA [83, 84].

Whilst this study relates to the mental health of adults it has been shown that cannabis use is linked with adverse mental health outcomes in offspring of exposed populations including autism and ADHD-like changes [83–94]. This important datum further amplifies the significance of the present investigation into the cross-generational context.

Hence taken together these data overall clearly indicate not only that increased cannabis use is causally associated with adverse mental health outcomes at the statistical and epidemiological level, but that multiple biological pathways exist to explain the causal relationship mechanistically. Indeed data in this report indicates that the cannabis-mental illness relationship fulfills all ten of Hill’s criteria for causal relationships [55], in addition to the unequivocal demonstration of very close relationships across space and time and the results of the causal inferential techniques employed.

### Strengths and limitations

Our study has a number of strengths and limitations. Its strengths include investigation of what we believe to be the best most carefully geospatially and temporally defined dataset in the world which measures both drug use and mental health outcomes in a synchronized and coordinated manner. Also we believe that the application of modern advanced geospatial analysis to these public health problems is also new and novel and innovative. The limitations of this study relate mainly to its ecological design. For example we had to estimate local use of cannabis by ethnic origin as substate estimates were not available. Similarly individual respondent data from the survey is not available outside of dedicated US research centres and it is clear that access to such data would increase the power of the present investigation. We feel therefore that while the present analysis represents an important contribution to the literature in the field it also provides a strong impetus for further research.

### Generalizability

Given that NSDUH is conducted carefully in a nationally representative manner of the non-institutionalized adult US population the present results are likely to be generalizable to other developed nations. Moreover as it appears that the geospatially observed trends are rooted in the biological processes and mechanisms, what we are seeing at the public health level reflects downstream pharmacological effects from altered biological processes occurring in human neurophysiology. We note that all five of the major racial groups investigated herein showed significant statistical relationships with mental health metrics suggesting cross-racial effects.

## Conclusions

Our interpretation of these results is that all four of the adverse mental health outcomes mapped geotemporospatially by SAMHSA are linked upon formal geospatial analysis with the use of all four of the addictive drugs for which data was available. On testing of single domains of variables against serious mental illness only the drug group was significant, whilst median household income and racial profiling were not. After adjustment for the usual battery of ethnic, drug use and socioeconomic covariates, terms including cannabis were significantly linked with all four domains of mental ill-health from a high level of statistical significance, implying that the widespread deployment of cannabis and cannabinoids for primarily commercial motivations is likely to carry with it major negative mental health implications for the future. Inverse probability weighting was employed to transform data from a purely ecological observational data series to a formal pseudo-randomized design. Highly significant estimates and confidence intervals at inverse probability-weighted robust and mixed effects regression together with large e-Values clearly indicate that these results fulfil the criteria for causal relationships. These epidemiological relationships are consistent with numerous cellular and molecular mechanisms describing cannabis-related neurotoxicity.

We find these results to be of great concern not only for the public health community within the USA but also for the wider international community.

## Supplementary Information

**Additional file 1: Supplementary Table 1.** Overall Data by Year. **Supplementary Table 2.** Line Slopes for Cannabis: Mental Illness Relationships (Fig. 3). **Supplementary Table 3.** Ethnic Cannabis Consumption Indices **Supplementary Table 4.** Spatial Panel General Method of Moments Models (Spgm) by Variable Domain. **Supplementary Table 5.** Spatial Panel Random Error Maximum Likelihood Models (Spreml) by Variable Domain. **Supplementary Table 6.** Comparison of Spreml Model Error Structure – Log Likelihood Values. **Supplementary Table 7.** Deciles of Cannabis Use and Modelled Serious Mental Illness. **Supplementary Table 8.** Mixed Effects Regression Results. **Supplementary Table 9.** Sensitivity Analysis – eValues. **Supplementary Table 10.** Analysis of Chi Squared Table for Trends by Legal Status.

**Additional file 2.**

**Additional file 3.**

**Additional file 4.**

**Additional file 5.**

**Additional file 6.**

**Additional file 7.**

**Additional file 8.**

## Data Availability

No permissions are required to access the data which was used and collated in this study, e.g. NSDUH study. Data including shapefiles and R programming script is made publicly available on the Mendeley Data Archive at this URL: 10.17632/gyckst6rx8.1 . The original SAMHSA shapefiles may be found at https://www.samhsa.gov/data/report/2014-2016-nsduh-substate-region-shapefile and https://www.samhsa.gov/data/report/2012-2014-nsduh-substate-region-shapefile.

## Abbreviations

Am: -American (Ethnicity)
ACS: American Community Survey
AFE: Attributable Fraction in the Exposed
AFP: Attributable Fraction in the Population
CRAN: Comprehensive R Archive Network
EPSG: European Petroleum Survey Group
e-Value: Expected Value
KKP: Kapoor, Kelejian and Prucha
LCEDI: Local Cannabis Ethnic Daily Index
LCEDPI: Local Cannabis Ethnic Daily Potency Index
LCEPI: Local Cannabis Ethnic Potency Index
MH: Mental Health
MHY: Median Household Income
NH-: Non-Hispanic
NSDUH: National Survey of Drug Use and Health
PR: Prevalence Ratio
SAMHSA: Substance Abuse and Mental Health Services Administration
SMI: Serious Mental Illness
spgm: Spatial Panel General Moments
spml: Spatial Panel Maximum Likelihood
spreml: Spatial Panel Random Effects Maximum Likelihood
USA: United States of America
