# Supplementary material for: Co-occurrence across time and space of drug- and cannabinoid- exposure and adverse mental health outcomes in the National Survey of Drug Use and Health: combined geotemporospatial and causal inference analysis
Source: BMC Public Health. 2020 Nov 4;20:1655. doi: 10.1186/s12889-020-09748-5 (PMC7640473; doi:10.1186/s12889-020-09748-5)
Supplement: Supplementary file 1 — Additional file 1: Supplementary Table 1. Overall Data by Year. Supplementary Table 2. Line Slopes for Cannabis: Mental Illness Relationships (Fig. 3). Supplementary Table 3. Ethnic Cannabis Consumption Indices Supplementary Table 4. Spatial Panel General Method of Moments Models (Spgm) by Variable Domain. Supplementary Table 5. Spatial Panel Random Error Maximum Likelihood Models (Spreml) by Variable Domain. Supplementary Table 6. Comparison of Spreml Model Error Structure – Log Likelihood Values. Supplementary Table 7. Deciles of Cannabis Use and Modelled Serious Mental Illness. Supplementary Table 8. Mixed Effects Regression Results. Supplementary Table 9. Sensitivity Analysis – eValues. Supplementary Table 10. Analysis of Chi Squared Table for Trends by Legal Status. [file 12889_2020_9748_MOESM1_ESM.docx]

**Supplementary Tables**

| **Table** | **Subject** | **Page** |
| --- | --- | --- |
|  |  |  |
| Supplementary Table 1 | Data Comparison by Year | 2 |
| Supplementary Table 2 | Regression Slopes of Lines | 4 |
| Supplementary Table 3 | Cannabis Use - Ethnicity Interactions | 5 |
| Supplementary Table 4 | Spgm Model Outputs by Variable Domain | 6 |
| Supplementary Table 5 | Spreml Model Outputs by Variable Domain | 8 |
| Supplementary Table 6 | Comparing Spreml Models | 10 |
| Supplementary Table 7 | Deciles of Cannabis Use and Serious Mental Illness | 11 |
| Supplementary Table 8 | Mixed Effects Model Regression Results | 12 |
| Supplementary Table 9 | Sensitivity Analysis - eValues | 13 |
| Supplementary Table 10 | Trend Analysis - Legal Status | 15 |

**Supplementary Table 1.: Overall Data by Year**

| **Parameter** | **Year** | | **Standardized Mean Difference** | **P-Value** |
| --- | --- | --- | --- | --- |
|  | **2011** | **2015** |  |  |
|  |  |  |  |  |
| N | 393 | 393 |  |  |
| Last Month Cigarette Use | 23.94 (21.12, 26.47) | 21.47 (18.23, 24.14) | 0.562 | <0.001 |
| Abuse or Dependence on Alcohol | 6.82 (5.97, 7.62) | 5.96 (5.35, 6.59) | 0.696 | <0.001 |
| Last Month Cannabis Use | 6.66 (5.27, 8.22) | 7.96 (6.59, 10.15) | 0.628 | <0.001 |
| Last Year Cannabis Use | 11.22 (9.29, 13.48) | 12.61 (10.75, 16.05) | 0.528 | <0.001 |
| Last Year Cocaine Use | 1.52 (1.27, 1.88) | 1.58 (1.23, 2.09) | 0.196 | 0.172 |
| Any Mental Illness, Last Year | 18.47 (17.41, 19.73) | 18.59 (17.44, 19.96) | 0.035 | 0.451 |
| Major Depressive Episode, Last Year | 6.81 (6.27, 7.41) | 6.94 (6.25, 7.37) | 0.015 | 0.625 |
| Serious Mental illness Last Year | 4.15 (3.70, 4.64) | 4.32 (3.89, 4.75) | 0.246 | <0.001 |
| Suicidal Thoughts,. Last Year | 3.83 (3.63, 4.17) | 4.13 (3.76, 4.46) | 0.542 | <0.001 |
| Cauc.Am_Percentage | 0.82 (0.67, 0.91) | 0.81 (0.67, 0.90) | 0.043 | 0.478 |
| Afric.Am_Percentage | 0.06 (0.02, 0.16) | 0.06 (0.02, 0.15) | 0.001 | 0.837 |
| Asian_Percentage | 0.02 (0.01, 0.04) | 0.02 (0.01, 0.04) | 0.056 | 0.164 |
| AIAN_Percentage | 0 (0.00, 0.01) | 0 (0.00, 0.01) | 0.016 | 0.974 |
| Hispanic_Percentage | 0.07 (0.03, 0.15) | 0.07 (0.04, 0.15) | 0.053 | 0.237 |
| Nat.Haw.Pac.Is.Am_Percentage | 0 (0.00, 0.00) | 0 (0.00, 0.00) | 0.009 | 0.019 |
| Median.Household.Income | 48901.72 (42799.56, 55991.94) | 52668.72 (46264.90, 61467.69) | 0.364 | <0.001 |
| Cauc.AmDaily.Cannabis | 0.19 (0.19, 0.19) | 0.23 (0.23, 0.23) | Not Defined | <0.001 |
| Afric.AmDaily.Cannabis | 0.22 (0.22, 0.22) | 0.32 (0.32, 0.32) | Not Defined | <0.001 |
| HispanicDaily.Cannabis | 0.16 (0.16, 0.16) | 0.18 (0.18, 0.18) | Not Defined | <0.001 |
| AsianDaily.Cannabis | 0.06 (0.06, 0.06) | 0.06 (0.06, 0.06) | Not Defined | <0.001 |
| AIANDaily.Cannabis | 0.2 (0.20, 0.20) | 0.39 (0.39, 0.39) | Not Defined | <0.001 |
| Nat.Haw.Pac.Is.AmDaily.Cannabis | 0.15 (0.15, 0.15) | 0.18 (0.18, 0.18) | Not Defined | <0.001 |
| Cauc.AmDaily.Cannabis.Local | 1.25 (0.99, 1.55) | 1.85 (1.53, 2.36) | 1.197 | <0.001 |
| Afric.AmDaily.Cannabis.Local | 1.49 (1.18, 1.83) | 2.55 (2.11, 3.25) | 1.559 | <0.001 |
| HispanicDaily.Cannabis.Local | 1.09 (0.86, 1.35) | 1.44 (1.19, 1.84) | 0.901 | <0.001 |
| AsianDaily.Cannabis.Local | 0.37 (0.29, 0.46) | 0.45 (0.37, 0.57) | 0.634 | <0.001 |
| AIANDaily.Cannabis.Local | 1.36 (1.08, 1.68) | 3.12 (2.58, 3.97) | 2.159 | <0.001 |
| Nat.Haw.Pac.Is.AmDaily.Cannabis.Local | 1.02 (0.81, 1.26) | 1.47 (1.22, 1.87) | 1.123 | <0.001 |
| THC_Potency | 11.01 (11.01, 11.01) | 15 (15.00, 15.00) | Not Defined | <0.001 |
| Cauc.AmDaily.Cannabis.Local.THC.Potency | 13.79 (10.91, 17.02) | 27.78 (23.00, 35.42) | 1.909 | <0.001 |
| Afric.AmDaily.Cannabis.Local.THC.Potency | 16.36 (12.94, 20.19) | 38.23 (31.65, 48.75) | 2.197 | <0.001 |
| HispanicDaily.Cannabis.Local.THC.Potency | 12.01 (9.50, 14.82) | 21.6 (17.88, 27.54) | 1.666 | <0.001 |
| AsianDaily.Cannabis.Local.THC.Potency | 4.1 (3.24, 5.05) | 6.68 (5.53, 8.52) | 1.44 | <0.001 |
| AIANDaily.Cannabis.Local.THC.Potency | 14.98 (11.85, 18.49) | 46.73 (38.69, 59.58) | 2.657 | <0.001 |
| Nat.Haw.Pac.Is.AmDaily.Cannabis.Local.THC.Potency | 11.25 (8.90, 13.89) | 22.02 (18.23, 28.08) | 1.849 | <0.001 |

Non-parametric Wilcoxons’s Test

Data listed as Median (Interquartile Range, 25%, 75%).

**Supplementary Table 2.: Line Slopes for Cannabis: Mental Illness Relationships (Figure 3)**

| **Mental Syndrome** | **Parameters** | | | | | **Model** | | | |
| --- | --- | --- | --- | --- | --- | --- | --- | --- | --- |
|  | **Parameter** | **Estimate** | **Std. Error** | **t value** | **P-Value** | **Adj. R Squared** | **F** | **df** | **P-Value** |
|  |  |  |  |  |  |  |  |  |  |
| Any Mental Illness | Cannabis_Monthly | 0.0433 | 0.0156 | 2.772 | 0.0059 | 0.0215 | 7.681 | 1,303 | 0.0059 |
| Major Depressive Episode | Cannabis_Monthly | 0.0389 | 0.0173 | 2.250 | 0.0252 | 0.0132 | 5.062 | 1,303 | 0.0252 |
| Serious Mental Illness | Cannabis_Monthly | 0.0201 | 0.0254 | 0.791 | 0.4298 | -0.0012 | 0.625 | 1,303 | 0.4298 |
| Suicidal Ideation | Cannabis_Monthly | 0.0775 | 0.0165 | 4.692 | 4.1E-06 | 0.0647 | 22.02 | 1,303 | 4.1E-06 |

**Supplementary Table 3.: Ethnic Cannabis Consumption Indices**

| **Ethnicity** | **% Population Smoking Cannabis * Ethnic % Smoking Cannabis, LCEDI** | | | |
| --- | --- | --- | --- | --- |
|  | **2011** | **2015** | **Relative_Rise LCEDI** | **Cf_Mean LCEDI** |
| Caucasian-American | 1.32 | 2.04 | 1.55 | 1.33 |
| African-American | 1.57 | 2.81 | 1.79 | 1.54 |
| Hispanic-American | 1.15 | 1.59 | 1.38 | 1.19 |
| Asian-American | 0.39 | 0.49 | 1.25 | 1.08 |
| American Indian / Alaskan Native | 1.44 | 3.43 | 2.38 | 2.06 |
| Native Hawaiian / Pacific Islander | 1.08 | 1.62 | 1.50 | 1.29 |
|  |  |  |  |  |
| **Ethnicity** | **Previous * THC Potency Cannabis, LCEDPI** | | | |
|  | **2011** | **2015** | **Relative_Rise LCEDPI** | **Cf_Mean LCEDPI** |
| Caucasian-American | 14.6 | 30.6 | 2.10 | 0.94 |
| African-American | 17.3 | 42.1 | 2.43 | 1.09 |
| Hispanic-American | 12.7 | 23.8 | 1.87 | 0.84 |
| Asian-American | 4.33 | 7.36 | 1.70 | 0.76 |
| American Indian / Alaskan Native | 15.8 | 51.4 | 3.25 | 1.46 |
| Native Hawaiian / Pacific Islander | 11.9 | 24.2 | 2.03 | 0.91 |

**Supplementary Table 4.: Spatial Panel General Method of Moments Models (Spgm) by Variable Domain**

| **Instrumental Variables** | **Dependent Variable** | **Parameter** | **Estimate (95% C.I.)** | **P-Value** | **Para-meters** | **Value** | **P-Value** |
| --- | --- | --- | --- | --- | --- | --- | --- |
|  |  |  |  |  |  |  |  |
|  |  | Drugs |  |  |  |  |  |
|  |  | ***3-Way Interactive Models*** |  |  |  |  |  |
|  | smiyr | ***spreml(smiyr ~ cigmon * mrjmon * Alcohol_Abuse + cocyr + MHY + 5_Races)*** | | | | | |
| NHWhite_Score |  | Alcohol | -5.23 (-6.5--3.96) | 7.8E-16 | phi | 0.0052 | 0.9945 |
| NHBlack_Score |  | Cannabis: Alcohol | 2.34 (1.71-2.97) | 1.4E-13 | psi | 0.4514 | 0.1725 |
| Hispanic_Score |  | Cigarettes: Alcohol | 0.2 (0.14-0.26) | 6.9E-13 | rho | -0.1597 | 0.3187 |
| NHAsian_Score |  | Cannabis | -3.87 (-4.99--2.75) | 1.3E-11 | lambda | 0.2857 | 0.01012 |
| NHAIAN_Score |  | Cigarettes: Cannabis: Alcohol | -0.09 (-0.11--0.07) | 2.8E-11 |  |  |  |
|  |  | Cigarettes | -0.33 (-0.43--0.23) | 5.5E-11 |  |  |  |
|  |  | Cigarettes: Cannabis | 0.16 (0.1-0.22) | 3.9E-10 |  |  |  |
|  |  | Cocaine | -0.1 (-0.14--0.06) | 2.0E-06 |  |  |  |
|  |  |  |  |  |  |  |  |
|  |  |  |  |  |  |  |  |
|  | smiyr | Income |  |  |  |  |  |
| NHWhite_Score |  | ***spreml(smiyr ~ Median_Household_Income)*** | | | | | |
| NHBlack_Score |  | Median_Household_Income | -0.27 (-0.33--0.21) | < 2.2e-16 | phi | 0.2773 | NA |
| Hispanic_Score |  |  |  |  | psi | 0.4591 | NA |
| NHAsian_Score |  |  |  |  | rho | 0.6135 | 2.3E-08 |
| NHAIAN_Score |  |  |  |  | lambda | 0.5771 | < 2.2e-16 |
|  |  |  |  |  |  |  |  |
|  |  |  |  |  |  |  |  |
|  |  |  |  |  |  |  |  |
|  | smiyr |  |  |  |  |  |  |
| NHWhite_Score |  | Race |  |  |  |  |  |
| NHBlack_Score |  | ***spreml(smiyr ~ Cauc.Am + Afric.Am + Hisp.Am + Asian.Am + AIAN.Am)*** | | | | | |
| Hispanic_Score |  | White_Fraction | 0.16 (0.1-0.22) | 1.9E-09 | phi | 0.0576 | 0.9690 |
| NHAsian_Score |  | Hispanic_Fraction | -0.03 (-0.05--0.01) | 9.9E-06 | psi | 0.4578 | 0.4646 |
| NHAIAN_Score |  | Asian_Fraction | -0.04 (-0.06--0.02) | 1.1E-06 | rho | 0.4865 | 3.0E-08 |
|  |  | AIAN_Fraction | 0.02 (0-0.04) | 6.4E-04 | lambda | -0.2728 | 0.0129 |
|  |  |  |  |  |  |  |  |
|  |  |  |  |  |  |  |  |
|  |  |  |  |  |  |  |  |
|  |  |  |  |  |  |  |  |

Technical Notes:

phi: - Idiosyncratic component of the spatial error term

psi: - Individual time-invariant component of the spatial error term

rho: - Spatial autoregressive parameter

lambda: - Spatial autocorrelation coefficient

**Supplementary Table 5.: Spatial Panel Random Error Maximum Likelihood Models (Spreml) by Variable Domain**

| **General** | | **Parameters** | | | **Model** | | |
| --- | --- | --- | --- | --- | --- | --- | --- |
| **Instrumental Variables** | **Dependent Variable** | **Parameter** | **Estimate (95% C.I.)** | **P-Value** | **Para-meters** | **Value** | **P-Value** |
|  |  |  |  |  |  |  |  |
|  |  | Drugs |  |  |  |  |  |
|  |  | ***3-Way Interactive Models*** |  |  |  |  |  |
|  | smiyr | ***spreml(smiyr ~ cigmon * mrjmon * Alcohol_Abuse + cocyr + MHY + 5_Races)*** | | | | | |
| NHWhite_Score |  | Alcohol | -5.23 (-6.5--3.96) | 7.8E-16 | phi | 0.0052 | 0.9945 |
| NHBlack_Score |  | Cannabis: Alcohol | 2.34 (1.71-2.97) | 1.4E-13 | psi | 0.4514 | 0.1725 |
| Hispanic_Score |  | Cigarettes: Alcohol | 0.2 (0.14-0.26) | 6.9E-13 | rho | -0.1597 | 0.3187 |
| NHAsian_Score |  | Cannabis | -3.87 (-4.99--2.75) | 1.3E-11 | lambda | 0.2857 | 0.01012 |
| NHAIAN_Score |  | Cigarettes: Cannabis: Alcohol | -0.09 (-0.11--0.07) | 2.8E-11 |  |  |  |
|  |  | Cigarettes | -0.33 (-0.43--0.23) | 5.5E-11 |  |  |  |
|  |  | Cigarettes: Cannabis | 0.16 (0.1-0.22) | 3.9E-10 |  |  |  |
|  |  | Cocaine | -0.1 (-0.14--0.06) | 2.0E-06 |  |  |  |
|  |  |  |  |  |  |  |  |
|  |  |  |  |  |  |  |  |
|  | smiyr | Income |  |  |  |  |  |
| NHWhite_Score |  | ***spreml(smiyr ~ Median_Household_Income)*** | | | | | |
| NHBlack_Score |  | Median_Household_Income | -0.27 (-0.33--0.21) | < 2.2e-16 | phi | 0.2773 | NA |
| Hispanic_Score |  |  |  |  | psi | 0.4591 | NA |
| NHAsian_Score |  |  |  |  | rho | 0.6135 | 2.3E-08 |
| NHAIAN_Score |  |  |  |  | lambda | 0.5771 | < 2.2e-16 |
|  |  |  |  |  |  |  |  |
|  |  |  |  |  |  |  |  |
|  |  |  |  |  |  |  |  |
|  | smiyr |  |  |  |  |  |  |
| NHWhite_Score |  | Race |  |  |  |  |  |
| NHBlack_Score |  | ***spreml(smiyr ~ Cauc.Am + Afric.Am + Hisp.Am + Asian.Am + AIAN.Am)*** | | | | | |
| Hispanic_Score |  | White_Fraction | 0.16 (0.1-0.22) | 1.9E-09 | phi | 0.0576 | 0.9690 |
| NHAsian_Score |  | Hispanic_Fraction | -0.03 (-0.05--0.01) | 9.9E-06 | psi | 0.4578 | 0.4646 |
| NHAIAN_Score |  | Asian_Fraction | -0.04 (-0.06--0.02) | 1.1E-06 | rho | 0.4865 | 3.0E-08 |
|  |  | AIAN_Fraction | 0.02 (0-0.04) | 6.4E-04 | lambda | -0.2728 | 0.0129 |
|  |  |  |  |  |  |  |  |

Technical Notes:

phi: - Idiosyncratic component of the spatial error term

psi: - Individual time-invariant component of the spatial error term

rho: - Spatial autoregressive parameter

lambda: - Spatial autocorrelation coefficient

**Supplementary Table 6.: Comparison of Spreml Model Error Structure – Log Likelihood Values**

| **Model Error Structure** | **Log Lik.** | **Chi Squared** | **Dergrees of Freedom** | **P-Value Spatial Hausman Test v. Full Model** |
| --- | --- | --- | --- | --- |
|  |  |  |  |  |
| spreml.sem2srrelag$logLik | 653.3919 | - | - | - |
| spreml.sem2srre$logLik | 653.2144 | 1652.1 | 15 | <2.2E-16 |
| spreml.sem2sr$logLik | 653.2144 | 1652.1 | 15 | <2.2E-16 |
| spreml.sem2re$logLik | 653.2144 | 1644.3 | 15 | <2.2E-16 |
| spreml.semsrre$logLik | 653.2143 | 1343.7 | 15 | <2.2E-16 |
| spreml.semre$logLik | 652.3119 | 765.57 | 15 | <2.2E-16 |
| spreml.srre$logLik | 641.4081 | 363.12 | 15 | <2.2E-16 |
| spreml.re$logLik | 641.4081 | 363.12 | 15 | <2.2E-16 |
| spreml.sr$logLik | 641.4081 | 363.12 | 15 | <2.2E-16 |
| spreml.ols$logLik | 625.9929 | 329.57 | 15 | <2.2E-16 |

**Supplementary Table 7.: Deciles of Cannabis Use and Modelled Serious Mental Illness**

| **Deciles** | **Monthly Cannabis Use Decile Midpoint** | **Modelled Log (SMI Rate)** | **Modelled SMI Rate** |
| --- | --- | --- | --- |
|  |  |  |  |
| 1 | 3.79 | 1.3737 | 3.9498 |
| 2 | 5.32 | 1.5018 | 4.4897 |
| 3 | 6.84 | 1.5958 | 4.9323 |
| 4 | 8.36 | 1.6701 | 5.3127 |
| 5 | 9.86 | 1.7315 | 5.6493 |
| 6 | 11.40 | 1.7839 | 5.9530 |
| 7 | 12.95 | 1.8295 | 6.2311 |
| 8 | 14.45 | 1.8700 | 6.4883 |
| 9 | 15.95 | 1.9063 | 6.7283 |
| 10 | 17.50 | 1.9393 | 6.9537 |
| 11 | 19.05 | 1.9694 | 7.1666 |

**Supplementary Table 8.: Mixed Effects Regression Results**

| **Parameter** | **Estimate (95% C.I.)** | **P-Value** |
| --- | --- | --- |
|  |  |  |
| ***Additive Models*** |  |  |
| Cigarettes | 0.01 (0.01-0.01) | 1.1E-13 |
| Cannabis | 0.11 (0.07-0.15) | 1.5E-10 |
| Median Household Income | 0.04 (0.02-0.06) | 0.0003 |
| Cauc.Am | 0 (0.00-0.00) | 0.0016 |
| Hispanic | -0.01 (-0.03-0.01) | 0.0379 |
| Alcohol | -0.01 (-0.01--0.01) | 0.0009 |
| Asian.Am | -0.03 (-0.05--0.01) | 0.0003 |
| Afric.Am | -0.02 (-0.02--0.02) | 1.3E-05 |
|  |  |  |
| ***Interactive Models*** |  |  |
|  |  |  |
| Cigarettes: Cocaine | 0.08 (0.04-0.12) | 2.1E-05 |
| Cannabis: Cocaine | 0.82 (0.45-1.19) | 2.7E-05 |
| Cauc.Am | 0 (0.00-0.00) | 0.0030 |
| Cigarettes: Cannabis | 0.04 (0.02-0.06) | 0.0033 |
| Median Household Income | 0.03 (0.01-0.05) | 0.0135 |
| Alcohol: Cannabis | 0.13 (0.01-0.25) | 0.0283 |
| Cigarettes: Alcohol | 0.01 (-0.01-0.03) | 0.0451 |
| Cigarettes: Alcohol: Cannabis | 0 (0.00-0.00) | 0.0456 |
| Alcohol | -0.29 (-0.54--0.04) | 0.0225 |
| Cigarettes | -0.07 (-0.13--0.01) | 0.0151 |
| Cannabis | -0.93 (-1.58--0.28) | 0.0050 |
| Asian.Am | -0.03 (-0.05--0.01) | 0.0009 |
| Cocaine | -1.77 (-2.59--0.95) | 2.9E-05 |
| Cigarettes: Cannabis: Cocaine | -0.04 (-0.06--0.02) | 1.9E-05 |
| Afric.Am | -0.02 (-0.02--0.02) | 1.5E-06 |

**Supplementary Table 9: Sensitivity Analysis – eValues**

| **Parameter** | **Table** | **β-Est. (C.I.)** | **R.R. (95%C.I.)** | **eValues** |
| --- | --- | --- | --- | --- |
|  |  |  |  |  |
| ***Legal Status*** |  |  |  |  |
| SMI Legal v. Illegal | Results |  | 1.09 (1.04, 1.13) | 1.40, 1.24 |
| SMI Decriminalized v Illegal |  |  | 1.03.5 (1.034, 1.036) | 1.23, 1.22 |
|  |  |  |  |  |
| ***Mixed Effects Models*** |  |  |  |  |
| ***Additive*** |  |  |  |  |
| Cannabis | Suppl. Table 8 | 0.11 (0.07, 0.15) | 1.72 (1.46, 2.02) | 2.83, 2.29 |
|  |  |  |  |  |
| ***Interactive*** |  |  |  |  |
| Cannabis: Cocaine |  | 0.82 (0.45, 1.19) | 54.33 (8.62, 342.28) | 108.16, 16.73 |
| Cannabis: Tobacco |  | 0.04 (0.02, 0.06) | 1.22 (1.07, 1.39) | 1.74, 1.34 |
|  |  |  |  |  |
| ***Spatial Models*** |  |  |  |  |
| ***spreml*** |  |  |  |  |
| ***Any Mental Illness*** |  |  |  |  |
| Cannabis: Alcohol | Table 2 | 0.96 (1.55, 2.36) | 5.06E+10 (3.09E+08, 8.23E+12) | 1.01E+11, 6.19E+08 |
| Cannabis: Tobacco |  | 0.12 (0.09, 0.16) | 4.70 (3.12, 7.08) | 8.87, 5.69 |
|  |  |  |  |  |
| ***Major Depressive Illness*** |  |  |  |  |
| Cannabis: Alcohol | Table 2 | 2.12 (1.63, 2.62) | 1.36E+09 (1.04E+07, 1.78E+11) | 2.72E+09, 2.08E+08 |
| Cannabis: Tobacco |  | 0.14 (0.10, 0.18) | 4.08 (2.76, 6.05) | 7.64, 4.97 |
|  |  |  |  |  |
| ***Serious Mental Illness*** |  |  |  |  |
| Cannabis: Alcohol | Table 2 | 1.8447 (1.30, 2.39) | 7.02E+06 (6.78E+04, 7.28E+08) | 1.40E+07, 1.36E+05 |
| Cannabis: Tobacco |  | 0.1175 (0.07, 0.16) | 2.72 (1.86, 3.99) | 4.90, 3.13 |
|  |  |  |  |  |
|  |  |  |  |  |
| ***Suicidal Thoughts*** |  |  |  |  |
| Cannabis: Tobacco | Table 2 | 0.1102 (0.07, 0.15) | 3.18 (2.16, 4.70) | 5.83, 3.74 |
| Cannabis: Alcohol |  | 1.8076 (1.33, 2.28) | 1.55E+08 (1.33E+06, 1.83E+10) | 3.11E+08, 2.66E+06 |

**Supplementary Table 10.: Analysis of Chi Squared Table for Trends by Legal Status**

| **Mental Syndrome** | **Chi-squared** | **df** | **P-value** |  |
| --- | --- | --- | --- | --- |
|  |  |  |  |  |
|  |  |  |  |  |
| Any Mental Illness | 163.00 | 133 | 0.0395 |  |
| Major Depressive Episode | 163.00 | 162 | 0.4632 |  |
| Serious Mental Illness | 157.35 | 132 | 0.0654 |  |
| Suicidal Ideation | 163.00 | 133 | 0.0395 |  |
