# Supplementary material for: Co-occurrence across time and space of drug- and cannabinoid- exposure and adverse mental health outcomes in the National Survey of Drug Use and Health: combined geotemporospatial and causal inference analysis
Source: BMC Public Health. 2020 Nov 4;20:1655. doi: 10.1186/s12889-020-09748-5 (PMC7640473; doi:10.1186/s12889-020-09748-5)

Ethnicity by Substate Area, 2011 and 2015

Data: US Census Bureau via R::Tidycensus & Areal; Shapefile from SAMHSA, NSDUH

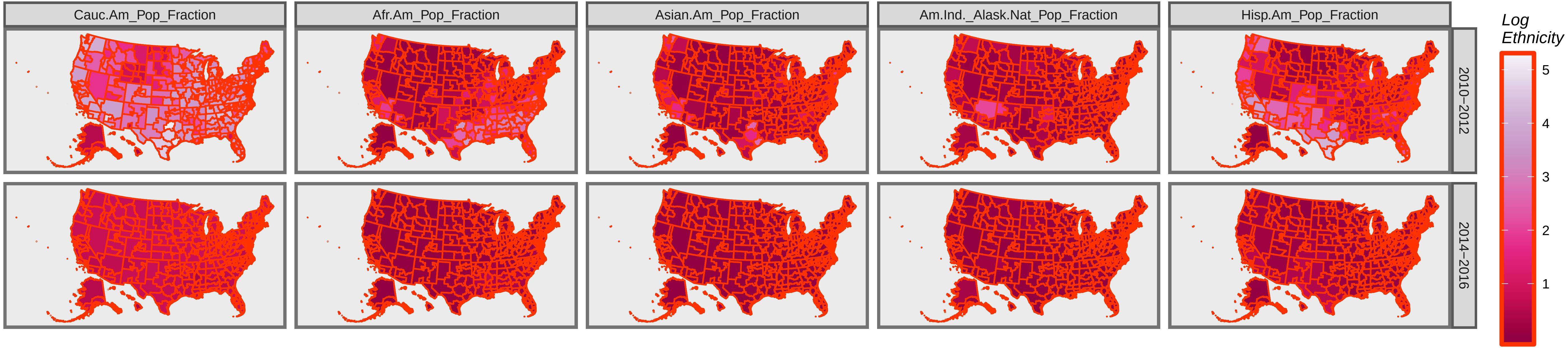

Supplement: Supplementary file 2 — Additional file 2. [file 12889_2020_9748_MOESM2_ESM.pdf]
