# Supplementary figures and images for: Co-occurrence across time and space of drug- and cannabinoid- exposure and adverse mental health outcomes in the National Survey of Drug Use and Health: combined geotemporospatial and causal inference analysis
Source: BMC Public Health. 2020 Nov 4;20:1655. doi: 10.1186/s12889-020-09748-5 (PMC7640473; doi:10.1186/s12889-020-09748-5)

Ethnic Cannabis Use x Frequency by Substate Area

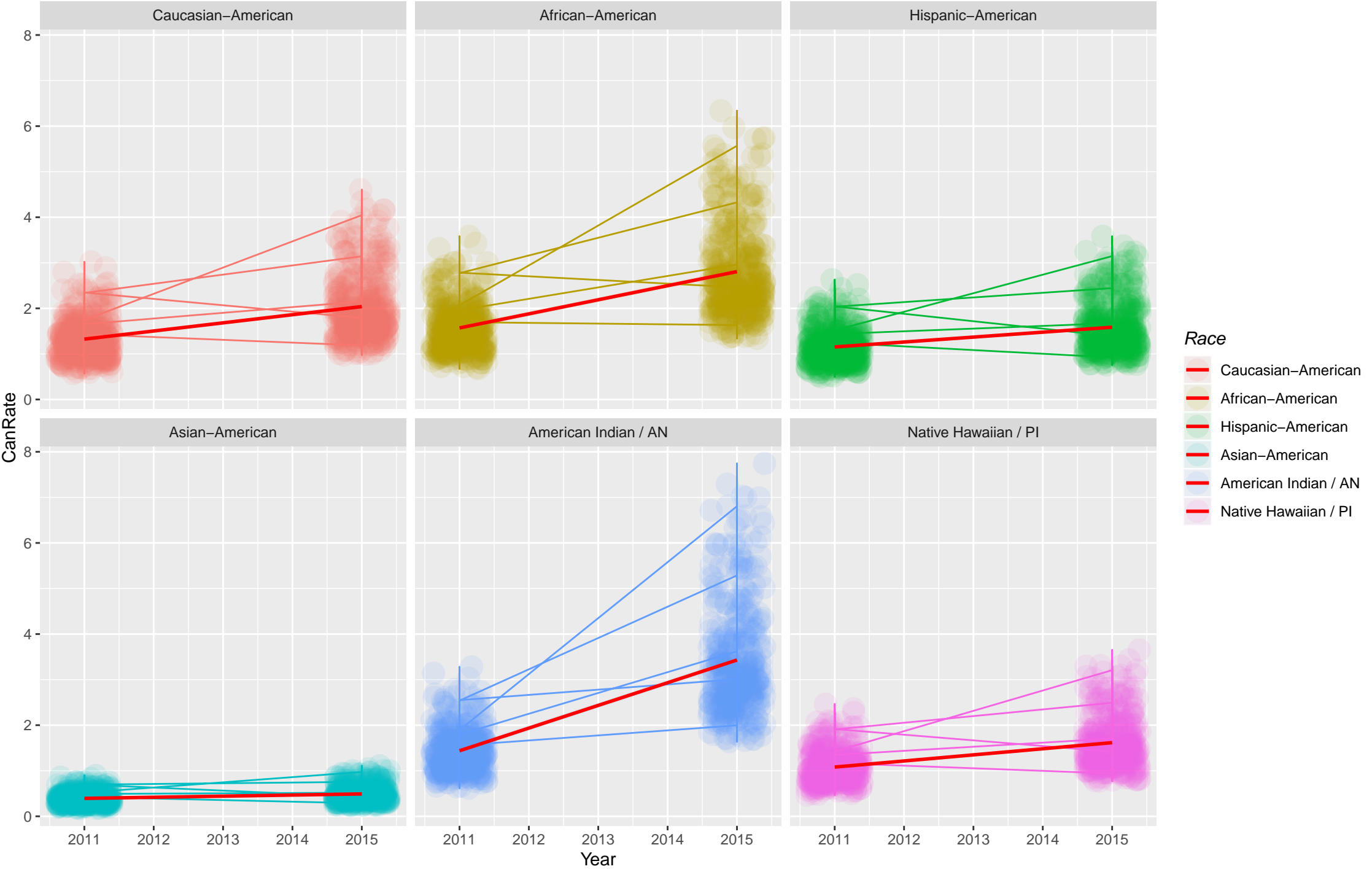

Supplement: Supplementary file 4 — Additional file 4. [file 12889_2020_9748_MOESM4_ESM.pdf]

# Ethnic Cannabis Use x Frequency x Cannabis Potency by Substate Area

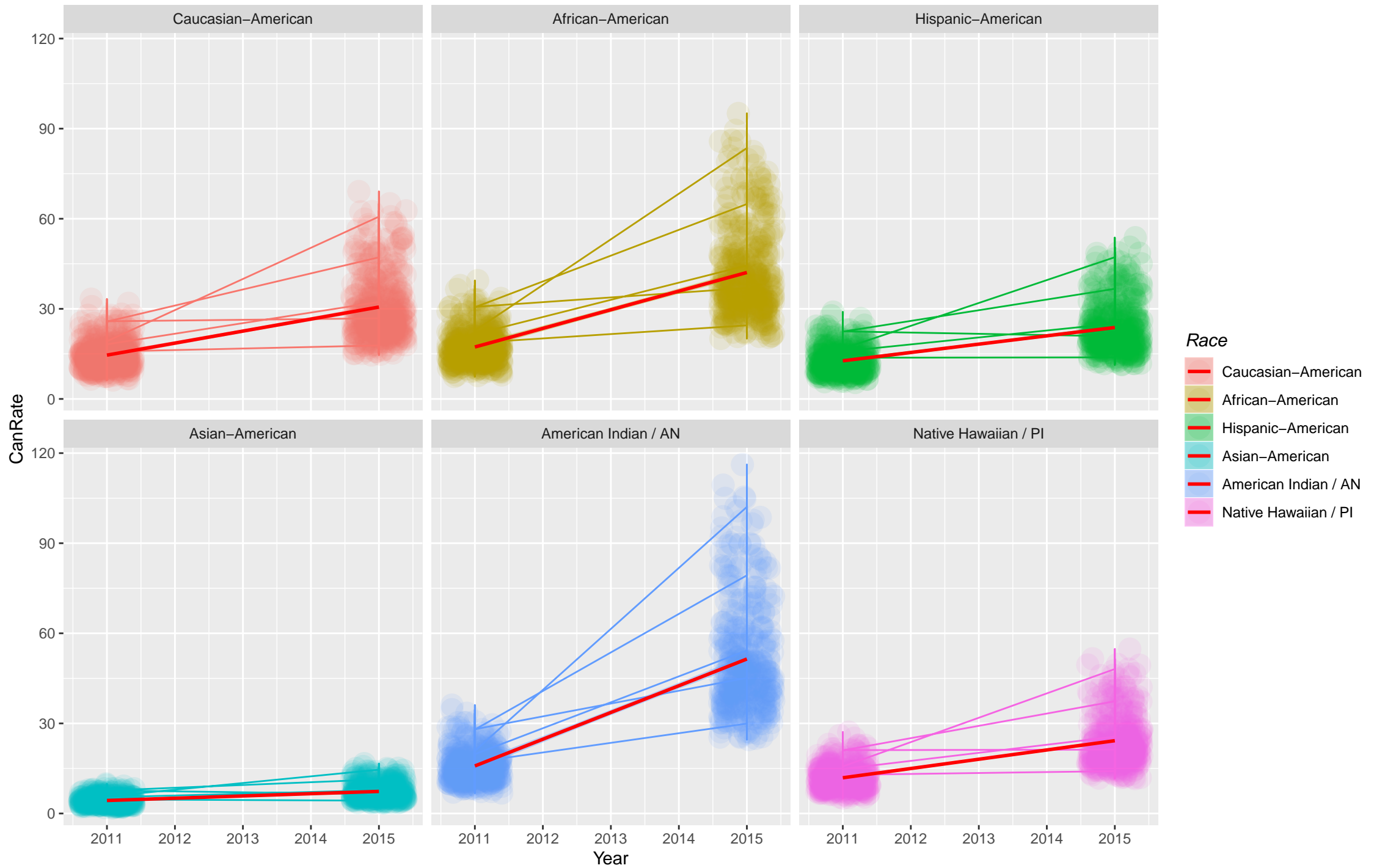

Supplement: Supplementary file 5 — Additional file 5. [file 12889_2020_9748_MOESM5_ESM.pdf]

# First Order Sub-State GAL Queen Weights (magenta) USCLong4444.nbb 1

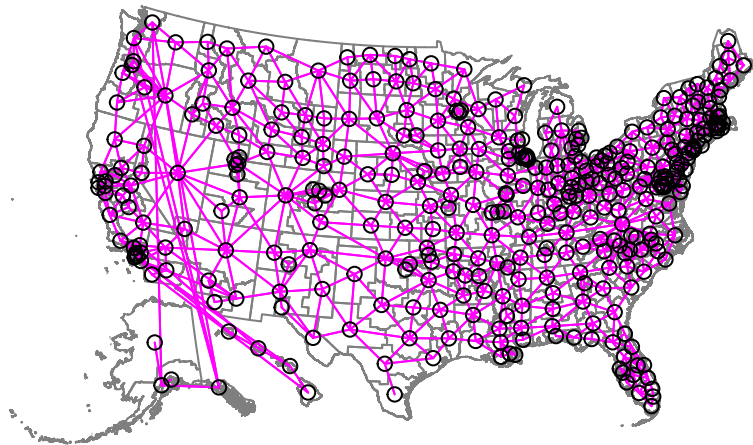

Supplement: Supplementary file 7 — Additional file 7. [file 12889_2020_9748_MOESM7_ESM.zip › SFigure 6B - Final LinksR4.pdf]

# Mental Illness by Cannabis Legal Status

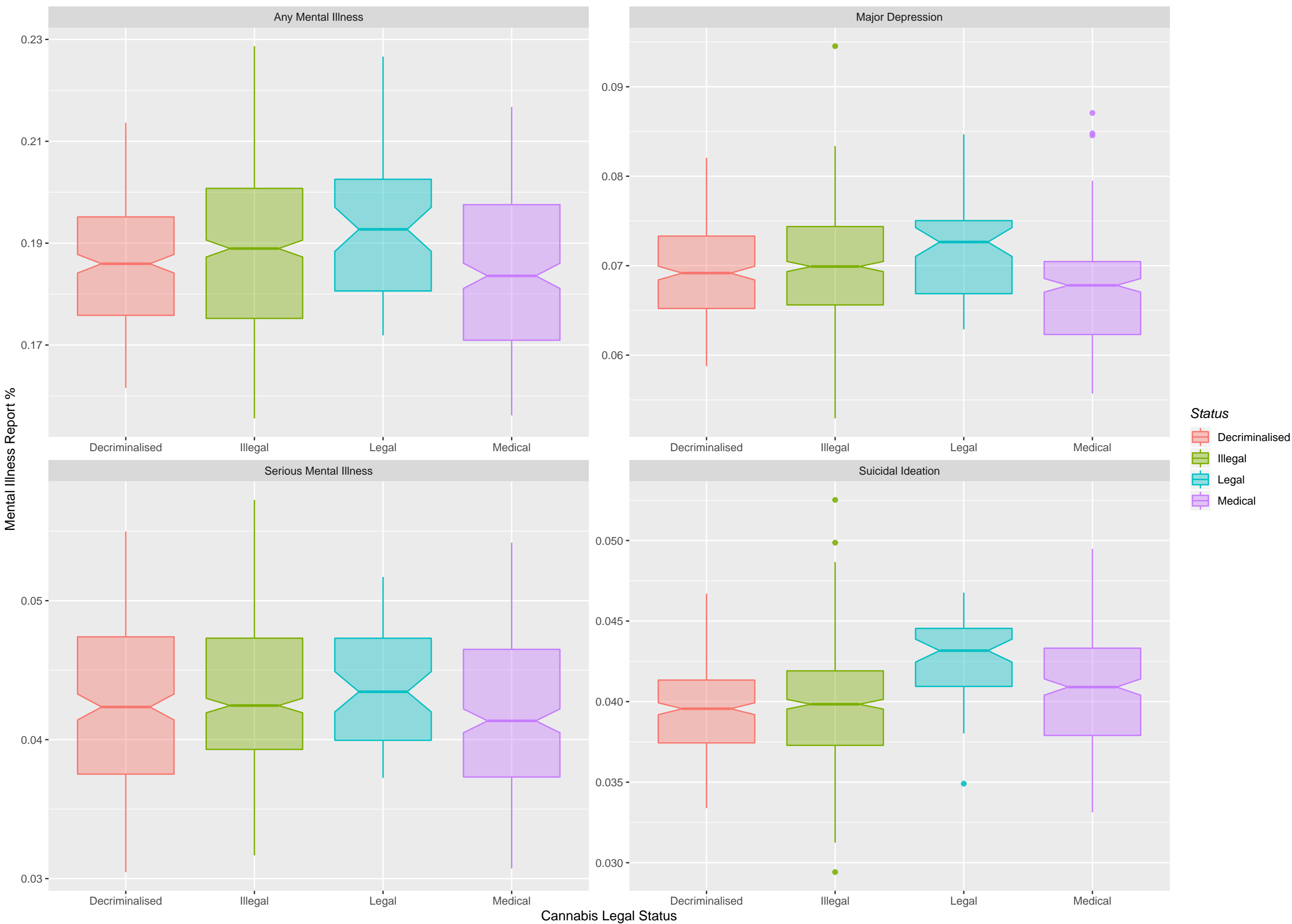

Supplement: Supplementary file 8 — Additional file 8. [file 12889_2020_9748_MOESM8_ESM.pdf]
