# Supplementary material for: Co-occurrence across time and space of drug- and cannabinoid- exposure and adverse mental health outcomes in the National Survey of Drug Use and Health: combined geotemporospatial and causal inference analysis
Source: BMC Public Health. 2020 Nov 4;20:1655. doi: 10.1186/s12889-020-09748-5 (PMC7640473; doi:10.1186/s12889-020-09748-5)
Supplement: Supplementary file 6 — Additional file 6. [file 12889_2020_9748_MOESM6_ESM.pdf]

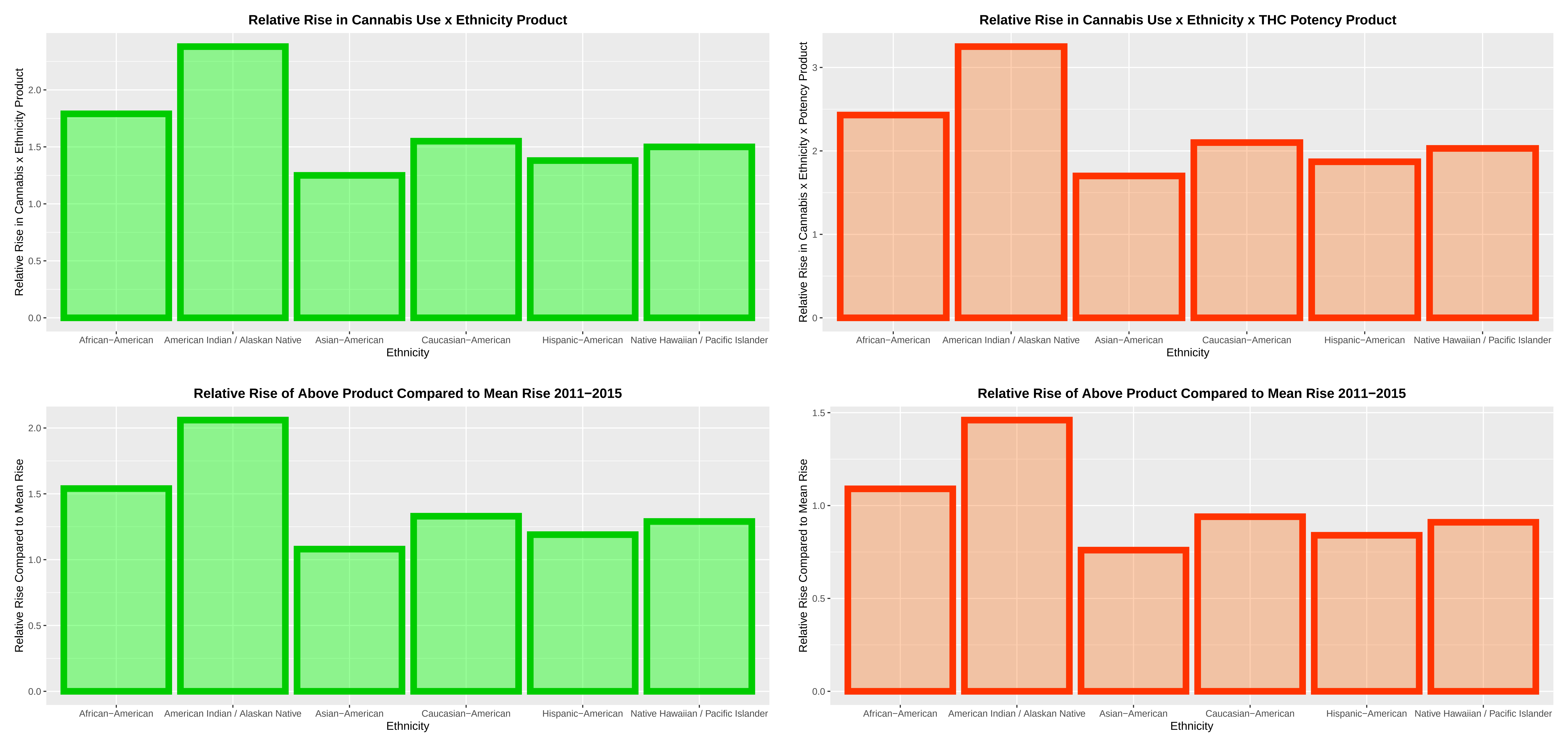
Relative Rise in Cannabis Use x Ethnicity Product
Relative Rise in Cannabis x Ethnicity Product
0.0
0.5
1.0
1.5
2.0
African−American
American Indian / Alaskan Native
Asian−American
Caucasian−American
Hispanic−American
Native Hawaiian / Pacific Islander
Ethnicity
Relative Rise in Cannabis Use x Ethnicity x THC Potency Product
Relative Rise in Cannabis x Ethnicity x Potency Product
0
1
2
3
African−American
American Indian / Alaskan Native
Asian−American
Caucasian−American
Hispanic−American
Native Hawaiian / Pacific Islander
Ethnicity
Relative Rise of Above Product Compared to Mean Rise 2011−2015
Relative Rise Compared to Mean Rise
0.0
0.5
1.0
1.5
2.0
African−American
American Indian / Alaskan Native
Asian−American
Caucasian−American
Hispanic−American
Native Hawaiian / Pacific Islander
Ethnicity
Relative Rise of Above Product Compared to Mean Rise 2011−2015
Relative Rise Compared to Mean Rise
0.0
0.5
1.0
1.5
African−American
American Indian / Alaskan Native
Asian−American
Caucasian−American
Hispanic−American
Native Hawaiian / Pacific Islander
Ethnicity
