# Supplementary material for: Co-occurrence across time and space of drug- and cannabinoid- exposure and adverse mental health outcomes in the National Survey of Drug Use and Health: combined geotemporospatial and causal inference analysis
Source: BMC Public Health. 2020 Nov 4;20:1655. doi: 10.1186/s12889-020-09748-5 (PMC7640473; doi:10.1186/s12889-020-09748-5)

SF6 - Geospatial Linkages and Weights

A

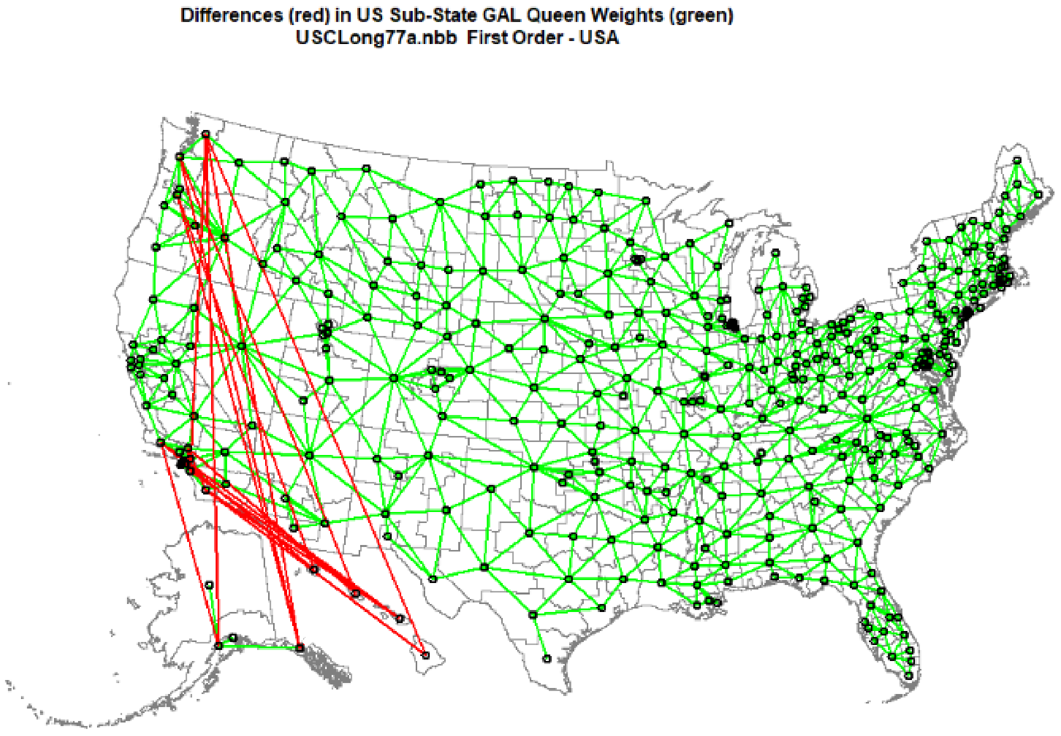

B

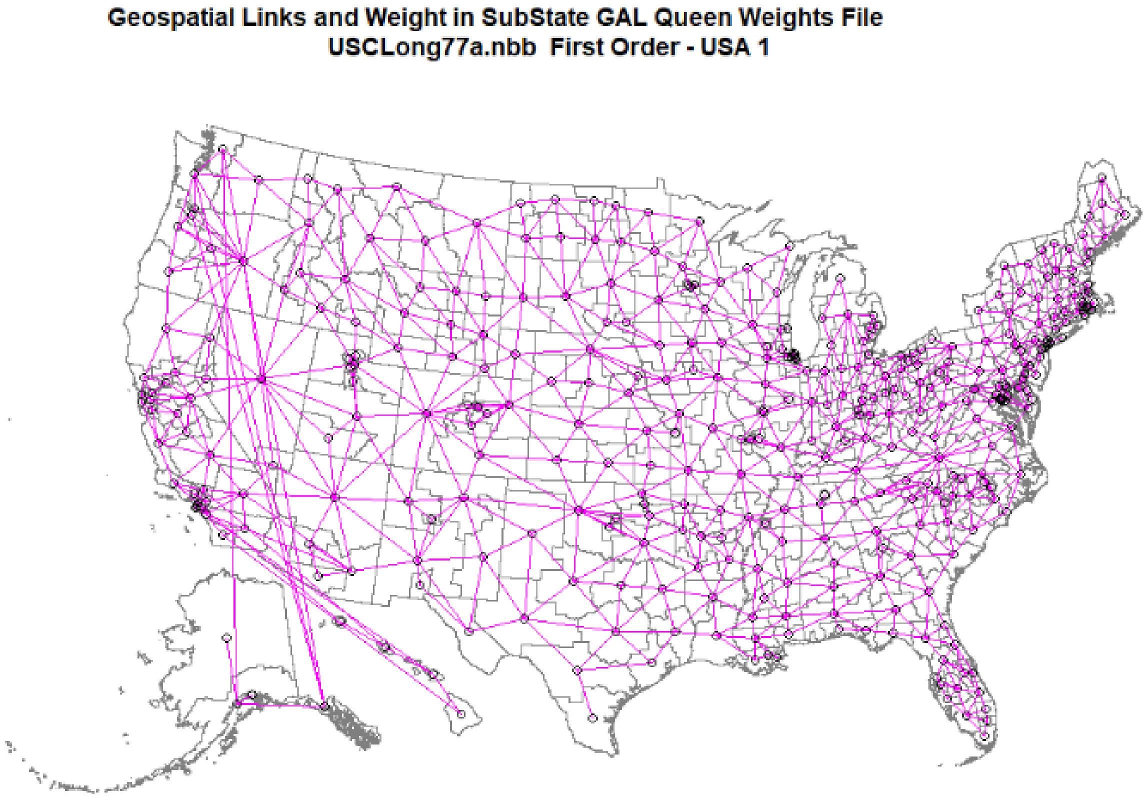

Supplement: Supplementary file 7 — Additional file 7. [file 12889_2020_9748_MOESM7_ESM.zip › SFigure 6 - Spatial LinksR4.pdf]
